# Comparison of Receptor–Ligand Restraint Schemes for Alchemical Absolute Binding Free Energy Calculations

**DOI:** 10.1021/acs.jctc.3c00139

**Published:** 2023-06-07

**Authors:** Finlay Clark, Graeme Robb, Daniel J. Cole, Julien Michel

**Affiliations:** † EaStCHEM School of Chemistry, University of Edinburgh, David Brewster Road, Edinburgh EH9 3FJ, United Kingdom; ‡ Oncology R&D, AstraZeneca, Cambridge CB4 0WG, United Kingdom; ¶ School of Natural and Environmental Sciences, Newcastle University, Newcastle upon Tyne NE1 7RU, United Kingdom

## Abstract

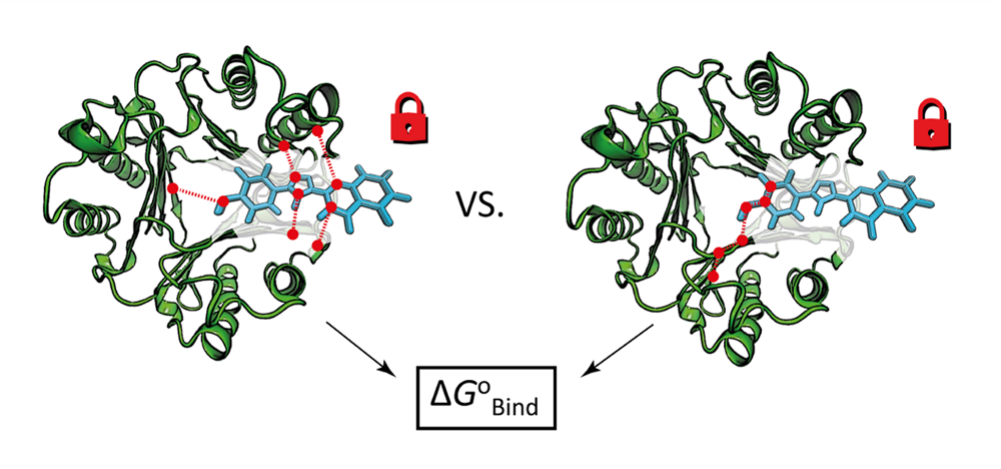

Alchemical absolute binding free energy calculations are of increasing interest in drug discovery. These calculations require restraints between the receptor and ligand to restrict their relative positions and, optionally, orientations. Boresch restraints are commonly used, but they must be carefully selected in order to sufficiently restrain the ligand and to avoid inherent instabilities. Applying multiple distance restraints between anchor points in the receptor and ligand provides an alternative framework without inherent instabilities which may provide convergence benefits by more strongly restricting the relative movements of the receptor and ligand. However, there is no simple method to calculate the free energy of releasing these restraints due to the coupling of the internal and external degrees of freedom of the receptor and ligand. Here, a method to rigorously calculate free energies of binding with multiple distance restraints by imposing intramolecular restraints on the anchor points is proposed. Absolute binding free energies for the human macrophage migration inhibitory factor/MIF180, system obtained using a variety of Boresch restraints and rigorous and nonrigorous implementations of multiple distance restraints are compared. It is shown that several multiple distance restraint schemes produce estimates in good agreement with Boresch restraints. In contrast, calculations without orientational restraints produce erroneously favorable free energies of binding by up to approximately 4 kcal mol^–1^. These approaches offer new options for the deployment of alchemical absolute binding free energy calculations.

## 1 Introduction

The *in silico* prediction of protein–ligand binding affinities is an important problem in drug discovery. The ability to rapidly and accurately calculate affinities for arbitrary protein–ligand systems would allow the efficient prioritization of compounds for synthesis and testing, accelerating the hit-to-lead and lead optimization stages of drug discovery.^1^

Recent improvements in computing power and automation have brought this vision closer to realization.^2−6^ In particular, alchemical methods are ideally suited for application during the hit-to-lead and lead optimization stages of drug discovery,^1,7^ as well as in the later stages of virtual screening.^8^ Along with path-based methods,^9^ alchemical simulations form a class of exact (in the limit of complete sampling and a perfect description of the potential energy) methods based on molecular dynamics or Monte Carlo sampling which provide binding affinity predictions of greater accuracy than alternatives.^10^ Modern computing resources now support routine use of alchemical relative binding free energy (RBFE) calculations to add value during drug discovery campaigns.^1^

RBFE calculations avoid the computationally intractable challenge of converging unbiased simulations of ligand binding and unbinding by instead gradually interconverting two structurally similar ligands. Interconversion proceeds through unphysical “alchemical” intermediates and is done in both the bound and unbound states.^11^ Based on a thermodynamic cycle, the free energy differences for each step are summed to yield the difference in the free energy of binding between the ligands. RBFE calculations are used routinely, and protocols for their robust deployment have been researched in detail.^12−15^ However, as a result of the requirement for a common ligand core, binding pose, and binding site, the following valuable problems typically lie outside the scope of RBFE calculations:^8,16^

(1) Calculating the RBFEs of structurally dissimilar ligands to a common target.
(2) Calculating RBFEs of the same ligand to the same protein with different binding poses.
(3) Calculating the RBFEs of the same ligand to different targets.
(4) Calculating the absolute binding free energy of a given ligand to a given target.

Alchemical absolute binding free energy (ABFE) calculations escape these limitations by following a more general thermodynamic cycle in which the ligand’s intermolecular interactions are completely turned off.^8^ In principle, these calculations can be used to calculate the binding free energies of structurally diverse molecules to varied targets, making them attractive for drug discovery. However, the alchemical ABFE framework presents challenges not encountered during an RBFE calculation. Restraints must be applied between the protein and ligand to avoid convergence issues such as those associated with the ligand “wandering” out of the binding site as its intermolecular interactions are removed.^17^ Restraints may also be required to avoid errors in the calculated binding free energies when the bound state is implicitly defined to include configurations where the ligand is anywhere in the entire simulation box relative to the receptor, as is the case when restraints are not used. However, these errors only affect weak binders (Section S1).

In general, it is nontrivial to select the optimum receptor–ligand restraints. Furthermore, ABFE calculations can be challenging to converge and therefore computationally costly because the ligand is completely removed.^18,19^ As a result, application studies still combine RBFE and ABFE, with ABFE applied more successfully to low molecular-weight compounds.^20^ Thus, there are barriers to the routine application of ABFE calculations.

The performance and accessibility of ABFE calculations would be improved if it was trivial to select receptor–ligand restraints which resulted in stable simulations and produced optimal convergence. While progress has been made in this direction with tools for automated or partially automated restraint selection,^3−5,18,21^ there is still no restraint type or selection method which completely solves this issue.

Receptor–ligand restraints of a variety of forms have been proposed. Following early work utilizing restraints on a single ligand atom,^22,23^ the first theoretically rigorous approach involving restraints on all of the external degrees of freedom (DoF) of the ligand was the Body Restraint Algorithm of Hermans and Wang.^24^ Later, the Virtual Bond Algorithm (VBA) of Boresch et al. was introduced,^17^ which involves restraining one distance, two bond angles, and three dihedral angles between six anchor points defined by the receptor and ligand. This provided a more convenient method to restrain the relative external DoF of the receptor and ligand, along with a simple analytical correction for releasing the restraints. The VBA has found widespread use and is often referred to as “Boresch restraints”.

However, despite their popularity, Boresch restraints suffer from a number of limitations and must be carefully applied to avoid numerical instabilities and sampling issues.^25^ For instance, if the anchor points are tied to the positions of highly flexible portions of the ligand or protein which do not strongly interact, then the restraints will be unable to maintain a binding pose similar to the restrained and interacting system, potentially leading to slow convergence of free energy estimates. Since only six relative external degrees of freedom can be restrained within this framework, there are limits on the extent to which ligand motions can be restricted. Thus, additional restraints on the intramolecular degrees of freedom of the ligand may be required to improve convergence for flexible ligands.^26^ Furthermore, if the restraints are poorly chosen, small changes in the Cartesian coordinates of the anchor points can result in large jumps in the six DoF defined in the VBA framework, resulting in the application of large forces, which can cause simulations to crash. This frequently occurs when sets of three contiguous anchor points approach collinearity,^3^ which can result in the application of large forces through the dihedral restraints.

Alternative restraint schemes have recently been proposed to address these issues. Fu et al. proposed a method in which the restrained six external DoF are derived by finding the optimal rotation of the ligand which minimizes its root-mean-square deviation (RMSD) with respect to a protein–ligand complex reference structure (after correcting for rotation and translation of the protein).^27^ By moving away from the six anchor points of the VBA, this was intended to simplify the selection of stable and efficient restraints. The “distance to bound configuration” (DBC) restraint is also intended to simplify restraint selection and to minimize the variance of free energy estimates for removing the ligand’s intermolecular interactions.^28−30^ This is achieved by directly restraining the RMSD of a subset of the ligand coordinates within the frame of reference of the binding site in order to optimally restrict the accessible configurational volume as the ligand intermolecular interactions are removed. However, because this scheme couples the internal and external degrees of freedom of the protein and ligand, there is no simple way to calculate the free energy of releasing the noninteracting ligand to the standard state. This necessitates a final stage to release the DBC restraints to a single harmonic restraint, for which the free energy of release is simple to calculate.

Another alternative to Boresch restraints is to restrain the distance between multiple receptor–ligand atom pairs. These restraints offer several advantages; for example, they can be intuitively selected to match native receptor–ligand interactions such as hydrogen bonds, thus closely mimicking the interacting state. This may accelerate convergence by tightly restricting ligand motion while intermolecular interactions are removed. Furthermore, these restraints do not suffer from the numerical instabilities inherent to the Boresch restraints scheme. Indeed, multiple distance restraints were used in an early study of the binding of biotin to streptavidin.^31^ However, the naive application of multiple distance restraints is theoretically incorrect, because they introduce coupling between the internal and external degrees of freedom of the protein and ligand, preventing the rigorous calculation of the free energy of releasing the noninteracting ligand.^32^ Despite this, a recent implementation has been described which relied on the assumption that the restraints were sufficiently weak that such coupling was negligible and that the free energy of turning on the restraints was close to zero.^33^ However, this was not verified, and the scheme has not been systematically compared to Boresch restraints.

**Figure 1:**
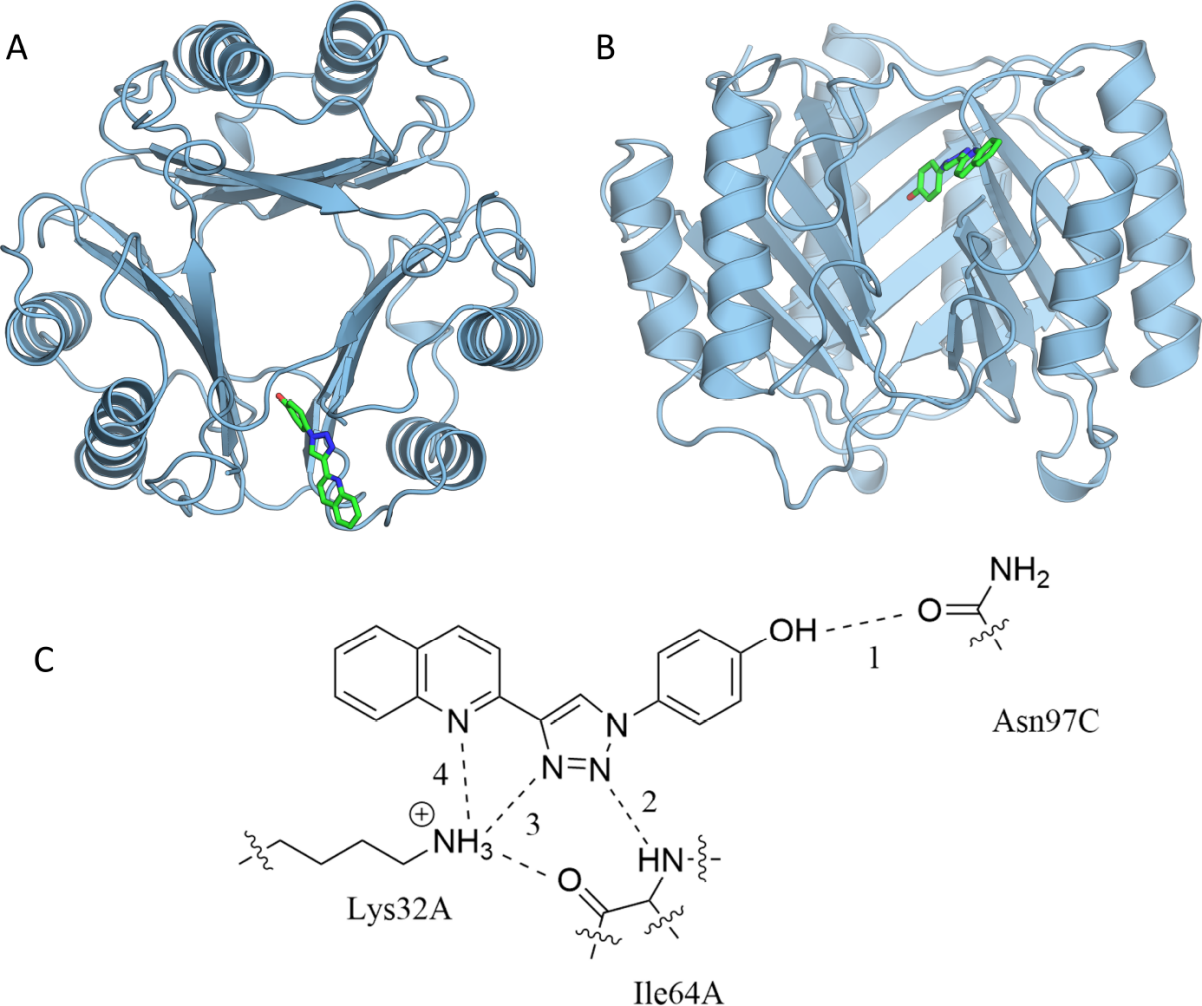
(A, B) MIF with MIF180 bound, rendered with PyMOL.^35^ (C) Hydrogen bonding interactions between MIF and MIF180 in the tautomerase active site. Redrawn from Qian et al.^34^

To address this, this study compares the absolute binding free energies obtained using Boresch restraints and different implementations of multiple distance restraints for a single ligand (MIF-180) binding to a single protein (human macrophage migration inhibitory factor, or MIF). This was suggested as a good model system by Qian et al.^34^ because MIF is a pharmaceutically relevant protein, but of moderate size (342 residues), and no major conformational changes occur in the protein upon ligand binding (Figure 1). In this work, the standard binding free energy of MIF-180 was calculated using multiple sets of Boresch restraint parameters. The results were compared to those produced by nonrigorous implementations of multiple distance restraints similar to that of Mendoza-Martinez et al.^33^ and two rigorous multiple distance restraint schemes: one inspired by Salari et al.^28^ and one newly developed.

## 2 Theory

### 2.1 Alchemical Absolute Binding Free Energy Calculations

**Figure 2:**
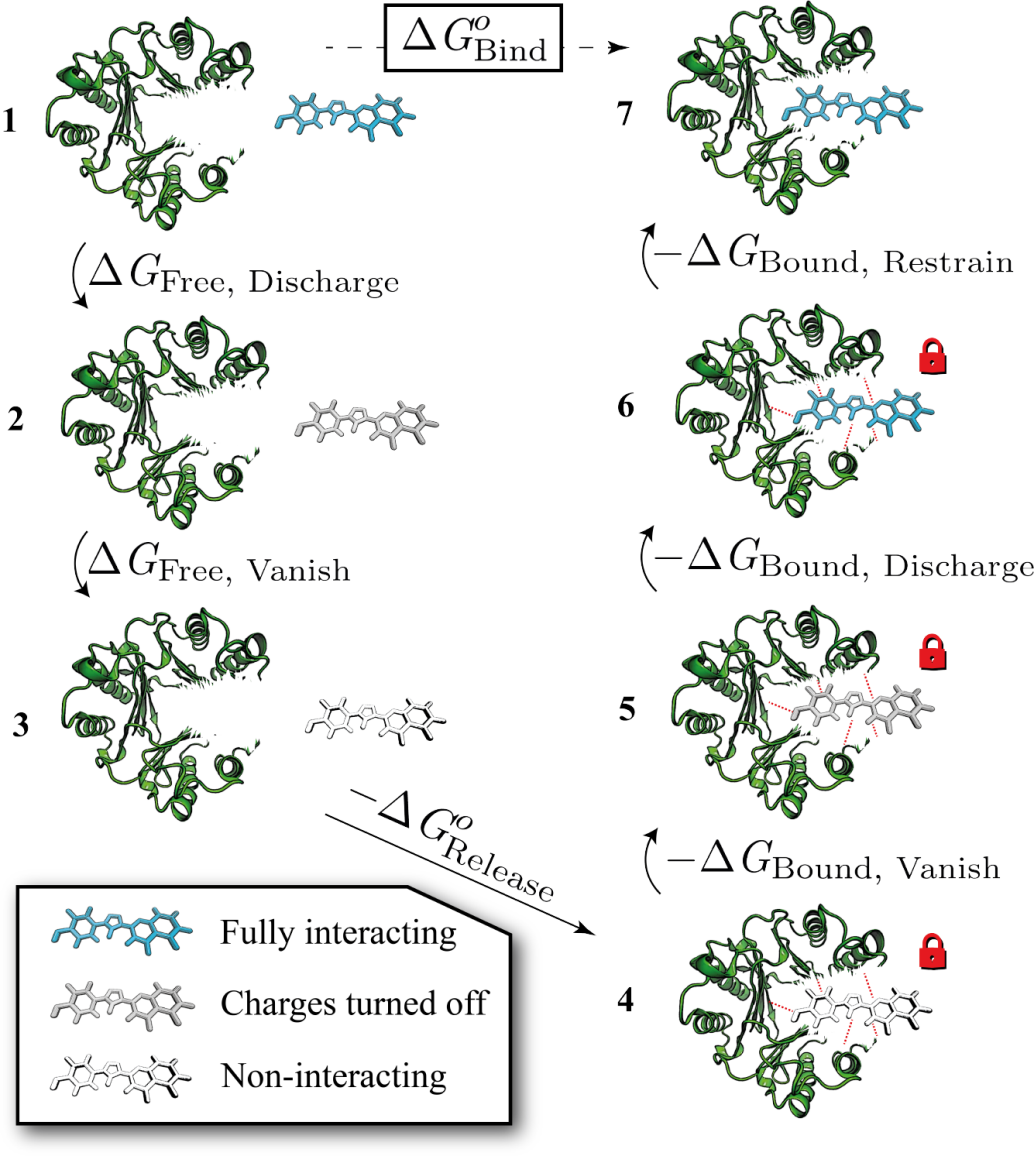
An alchemical thermodynamic cycle for the calculation of ABFEs. The red dashed lines indicate protein–ligand restraints. It is generally computationally intractable to obtain *ΔG*_Bind_^*o*^ by direct simulation, but the alchemical cycle allows *ΔG*_Bind_^*o*^ to be obtained through a series of states which are less challenging to sample at equilibrium. *ΔG*_Release_^*o*^ is calculated without simulation.

ABFEs can be computed using an alchemical cycle (Figure 2). The standard free energy of binding is calculated by adding up the terms around the cycle:
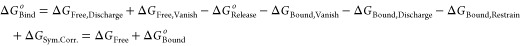
1where “Free” and “Bound” indicate that the ligand is in solution or in the receptor binding site. “Discharge” means removal of ligand Coulombic interactions. “Vanish” indicates removal of the ligand Lennard-Jones (LJ) interactions, and “Restrain” means introduction of intermolecular restraints between the receptor and ligand. Δ*G*_Release_^*o*^ is the correction for releasing the receptor–ligand restraints when the ligand has no intermolecular interactions, and *ΔG*_Sym.Corr._ accounts for symmetries broken by the introduction of restraints.^36^ The bound leg refers to all calculations where the ligand is in the binding site and includes the symmetry correction, and the free leg describes all calculations where the ligand is in solution.

When only the intermolecular components of the ligand nonbonded interactions (Coulombic or Lennard-Jones (LJ)) are removed, this is often termed “decoupling”, while “annihilation” may refer to removal of both the inter- and intramolecular components.^37^ However, the terminology of Gilson et al. is used here:^38^ “decoupling” denotes removal of of the ligand intermolecular interactions while enforcing receptor–ligand restraints, irrespective of how the intramolecular interactions are treated.

### 2.2 Receptor–Ligand Restraints

The free energy of releasing the restraint on the decoupled ligand is given by the ratio of configurational integrals
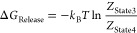
2where *k*_B_ is the Boltzmann constant, *T* is the temperature, and *Z*_State3_ and *Z*_State4_ are the configurational integrals for States 3 and 4, as defined in Figure 2. However, this result is dependent on the size of the water box in State 3, *V*_Box_. A more useful quantity is the standard free energy of binding
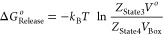
3which is independent of *V*_Box_. *V*^*o*^ = 1660 Å^3^ is the standard state volume. This ratio could be evaluated using a simulation in which the restrained decoupled ligand is completely released into the simulation box, but this would be slow to converge. Instead, this ratio must be simplified so that it can be evaluated without simulation. It can be shown (Section S2) that eq 3 can be written
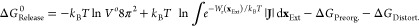
4where *W*_r_(**x**_Ext_) is the potential of mean force (PMF) of the receptor–ligand restraint energy with respect to the six relative receptor–ligand external DoF, **x**_Ext_. The form of the Jacobian determinant, |**J**|, depends on the coordinate transformation used to extract the relative external degrees of freedom from the internal degrees of freedom of the complex. *ΔG*_Preorg._ accounts for straining of the receptor and decoupled ligand when *W*_r_(**x**_Ext_) is at its minimum, while Δ*G*_Distort._ accounts for further distortion of the receptor and ligand when *W*_r_(**x**_Ext_) is not at its minimum.

For an arbitrary set of receptor–ligand restraints, there is no straightforward way to evaluate this expression. The standard solution to obtain *ΔG*_Release_^*o*^ is to select a set of receptor–ligand restraints for which the restraint energy, *U*_r_(**x**_Ext_), is a function of only the receptor–ligand relative external degrees of freedom. In this case, *ΔG*_Preorg._ = *ΔG*_Distort._ = 0, because the relative external coordinates of the decoupled ligand and receptor are uncorrelated with the internal DoF and *W*_r_(**x**_Ext_) = *U*_r_(**x**_Ext_). Restraints of this form are described as not coupling the internal and external degrees of freedom of the receptor and ligand. Intuitively, such restraints do no “squeeze” or “stretch” the receptor or decoupled ligand. The free energy of releasing these restraints is
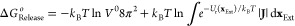
5which can be integrated directly. Thus, the ideal receptor–ligand restraints would not couple the internal and external degrees of freedom of the receptor and ligand, so that eq 5 is valid. The ideal restraints would also ensure optimal convergence of the bound stages. This might be achieved by mimicking the native receptor–ligand interactions as closely as possible,^16^ thus minimally perturbing the fully interacting complex while maximally restricting the accessible configurational volume during decoupling.^29^ However, the extent to which this can be achieved is limited when restraining only six DoF. Hence, it may be desirable to use restraints which do couple the these internal and relative external degrees of freedom in order to accelerate convergence of the decoupling calculations. In this case, the restraints which do couple the these degrees of freedom should be released through simulation to those which do not (and the associated free energy change accounted for), or some degree of error must be tolerated in the calculation of *ΔG*_Release_^*o*^. Finally, the ideal restraints would lack instabilities, and would be simple to select in an easily automatable manner.^4,27,39^

### 2.3 Boresch Restraints

**Figure 3:**
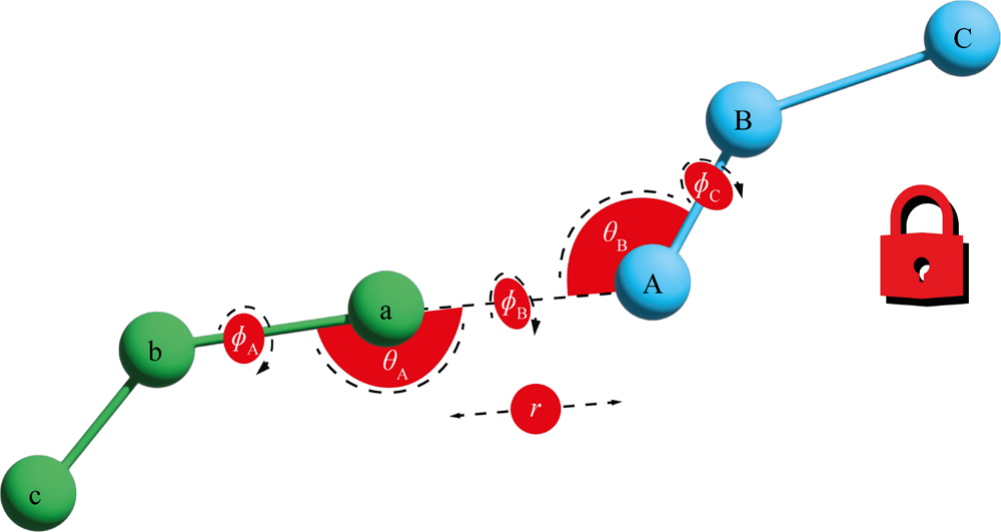
General form of Boresch restraints.^17^ Three anchor points (a, b, and c) are selected based on the receptor (green) coordinates and three (A, B, and C) are selected based on the ligand (blue) coordinates. The restrained six external degrees of freedom are defined as one distance (*r*), two bond angles (θ_A_ and θ_B_), and three dihedral angles (ϕ_A_, ϕ_B_, and ϕ_C_). In this diagram, the contiguous anchor points b, a, and A are close to collinear and therefore these restraints would be expected to be unstable.

Boresch restraints (Figure 3) only affect the six relative external degrees of freedom of the ligand with respect to the receptor, and do not couple the receptor and ligand internal and external degrees of freedom.^17^ As a result, eq 5 can be easily evaluated by numerical integration of the expression
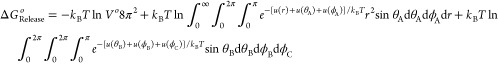
6where *u*(*x*) is the restraining potential applied to the degree of freedom *x*. These degrees of freedom are defined in Figure 3. The second term in eq 6 integrates over the position of anchor atom A with respect to the receptor coordinates, and does not depend on anchor atoms B and C. The third term integrates over the orientation of the ligand with respect to the receptor. Hence, Boresch restraints can be used to restrain only the position of the anchor point A with respect to the receptor, by setting *u*(θ_B_), *u*(ϕ_B_), and *u*(ϕ_C_) to 0.

Although it is common to use harmonic restraining potentials and to select the anchor points as atomic positions, these are not constraints of the framework. For example, periodic dihedral restraints can be used,^5^ and anchor points may be derived from multiple atomic positions or centers of mass.^34^ Harmonic restraints are used in this work. In this case, eq 6 can be evaluated analytically as
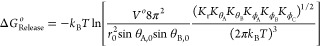
7where *K* denotes a force constant and 0 denotes an equilibrium value.^17^ This assumes that *r*, sin θ_A_, and sin θ_B_ can be taken out of the integrals in eq 6 and replaced by their equilibrium values.

As mentioned previously, when the anchor points are arranged so that large changes in the six DoF defined in the VBA framework result from small changes to the Cartesian coordinates of atoms, this can result in large forces and simulation crashes. To avoid such instabilities, anchor points must be carefully selected to avoid the collinearity of any three contiguous anchor points.

Algorithms have been proposed for the selection of Boresch restraints.^3,16,18,40−42^ At a minimum, these aim to select stable restraints based on the geometry of the complex, while more sophisticated methods aim to enhance convergence by directly (e.g., based on H-bonds) or indirectly (based on minimum total variance of the distance, angles, and dihedrals) mimicking strong receptor–ligand interactions based on a short unrestrained simulation. However, there is no obviously superior method which has been shown to guarantee selection of numerically stable restraints with optimal convergence properties.

### 2.4 Multiple Distance Restraints

**Figure 4:**
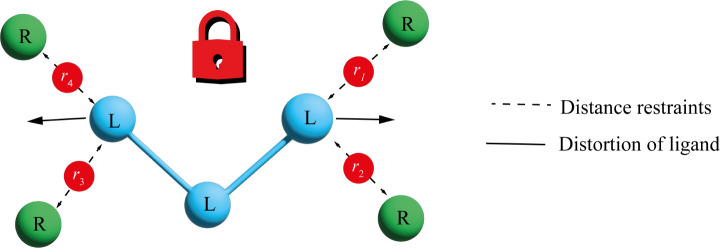
A ligand restrained using multiple distance restraints. Four distance restraints (*r*_1_–*r*_4_) are applied between pairs of atoms in the ligand (blue) and the receptor (green). Applying distance restraints between several receptor–ligand atom pairs can allow greater restriction of ligand movement than a typical six DoF restraint scheme. However, multiple distance restraints can couple the internal and external degrees of freedom of the receptor and ligand, as shown by the distortion of the ligand, preventing rigorous calculation of the free energy of releasing the decoupled ligand.

Restraints schemes based on multiple distance restraints do not suffer from the inherent instabilities of Boresch restraints, and allow the ligand to be restrained to a greater extent (Figure 4). In this work, harmonic or flat-bottomed distance restraints were used. Using the former, *U*_r_ is given by
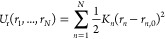
8and with the latter the total restraint energy is given by
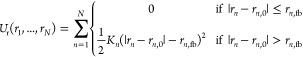
9where *r*_*n*_ is the distance between atoms in a restrained pair, *r*_*n*,0_ is the equilibrium distance, *r*_*n*,fb_ is the flat-bottomed radius, and *K*_*n*_ is the force constant for restrained pair *n. N* is the total number of restrained pairs.

**Figure 5:**
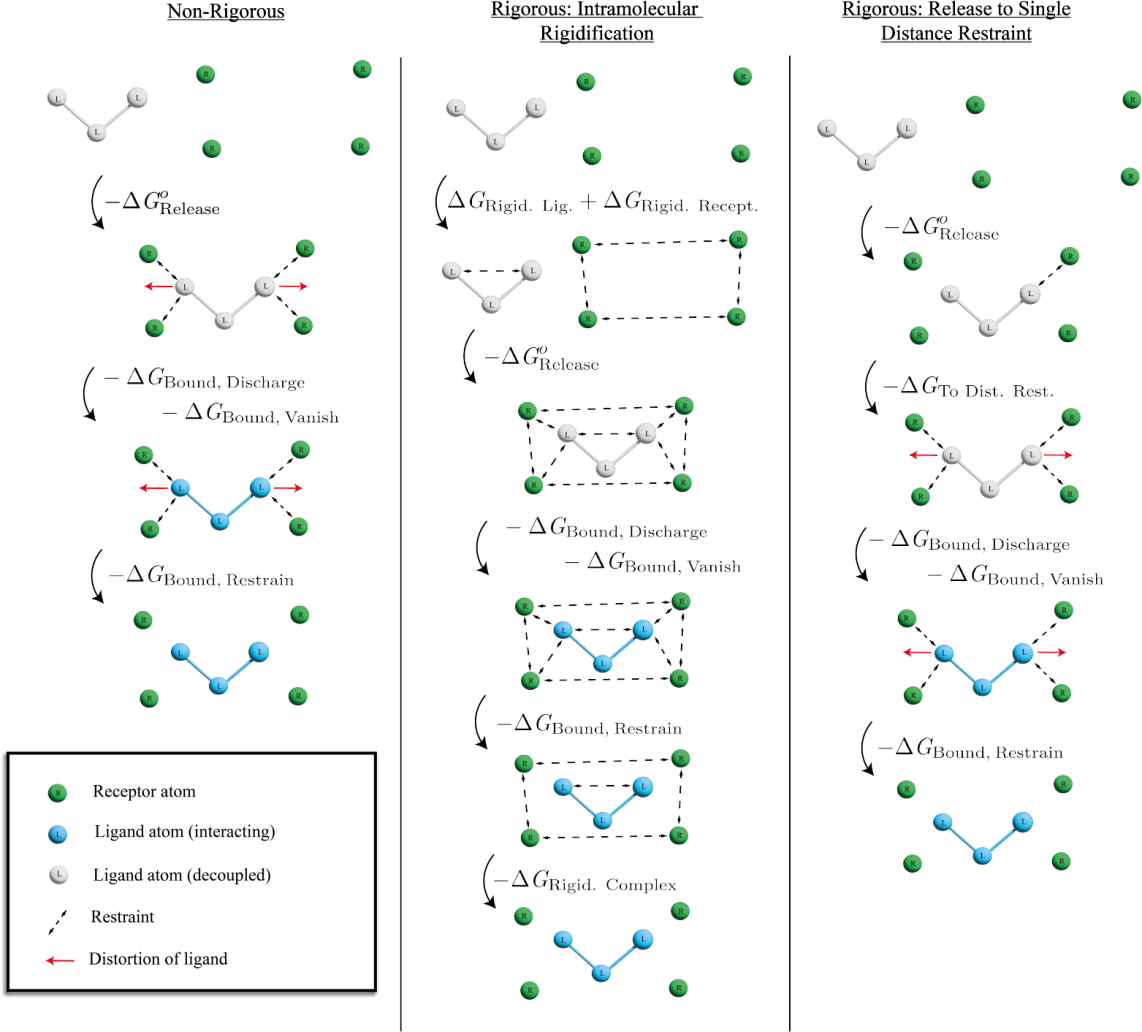
Multiple distance restraints schemes. From top to bottom, all schemes start in State 3 and progress to State 7 (Figure 2). For the example shown, the “naive” (nonrigorous) multiple distance restraint scheme may suffer from large systematic error due to distortion of the flexible ligand, which is not accounted for in the −*ΔG*_Release_^*o*^ stage. This is avoided in the other schemes by either applying strong intramolecular restraints, or releasing all but one distance restraint.

The main flaw of multiple distance restraints is that, applied naively, they may couple the internal and external degrees of freedom of the receptor and ligand, preventing the simplification of eq 4 to 5 and hence the calculation of *ΔG*_Release_^*o*^. Here, we investigate three approaches to circumvent this difficulty (Figure 5). The first is to implement multiple distance restraints in such a way that the error introduced is negligible (the “naive” approach). This is the basis for recent implementations in the Michel group,^33,43,44^ in which sets of relatively permissive flat-bottomed restraints were used. An approximate value for *ΔG*_Release_^*o*^ is calculated by numerical integration of
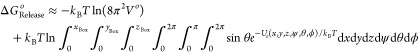
10where *x*_Box_, *y*_Box_, and *z*_Box_ are the side lengths of the simulation box, and *x*, *y*, *z*, ψ, θ, and ϕ are the Cartesian coordinates of the center of mass and the Euler angles of the ligand in the frame of reference of the receptor. This is evaluated by taking the average intramolecular coordinates of the anchor points from a simulation of State 4. The receptor and ligand are then assumed to be rigid, allowing *U*_r_(*x*, *y*, *z*, ψ, θ, ϕ) to be calculated by translating and rotating the ligand anchor points with respect to the receptor anchor points and evaluating *U*_r, Rigid_(*r*_1_, ..., *r*_*N*_) = *U*_r, Rigid_(*x*, *y*, *z*, ψ, θ, ϕ) at each point, where “Rigid” shows that the anchor points have been fixed to their average intramolecular positions.

This assumes that *ΔG*_Preorg._ = 0 (eq 4), and produces a bias toward more negative free energies of binding, because *ΔG*_Preorg._ ≥ 0. The error will be substantial when the average intramolecular positions of the anchor points for the decoupled complex are very different to those in the free ligand and receptor, or the restraints substantially restrict the conformational freedom of the receptor or ligand, thus enforcing substantial “preorganization”. The magnitude of the error would be expected to increase with the number and strength of restraints, and decreasing volume of the flat-bottom region. This is likely to be particularly problematic for systems where there are substantial changes in the conformations of the ligand and the binding site upon binding. In addition, the use of average positions assumes that *ΔG*_Distort._ = 0 and *U*_r, Rigid_(*x*, *y*, *z*, ψ, θ, ϕ) = *W*_r_(*x*, *y*, *z*, ψ, θ, ϕ). This produces a slight bias toward more positive free energies of binding, because a flexible system will distort to minimize the sum *W*_r_(*x*, *y*, *z*, ψ, θ, ϕ) + *ΔG*_Distort., Point_(*x*, *y*, *z*, ψ, θ, ϕ) ≤ *U*_r, Rigid_(*x*, *y*, *z*, ψ, θ, ϕ) for a given relative position and orientation. *ΔG*_Distort., Point_(*x*, *y*, *z*, ψ, θ, ϕ) is related to *ΔG*_Distort._ by integration over the six external DoF. Overall, neglecting flexibility is expected to result in an erroneously negative *ΔG*_Release_^*o*^ due to neglect of *ΔG*_Preorg._. Obtaining an accurate free energy of binding under these approximations depends upon the restraints being sufficiently permissive and there being no large changes in the average intramolecular coordinates of the anchor points during binding.

Here, we investigate an alternative scheme to eliminate these sources of error: applying intramolecular restraints to rigidify the anchor points. The free energy of applying and releasing these in the free and bound stages can be explicitly calculated with simulation. The restraints should be sufficiently strong to “preorganize” the system, and substantially stronger than the intermolecular restraints. This guarantees that *ΔG*_Preorg._ = *ΔG*_Distort._ = 0 and that *W*_r_(*x*, *y*, *z*, ψ, θ, ϕ) = *U*_r, Rigid_(*x*, *y*, *z*, ψ, θ, ϕ), rendering eq 10 rigorous.

An alternative rigorous approach to the implementation of multiple distance restraints is to evaluate the free energy change, *ΔG*_To Dist. Rest._, for releasing them to a single harmonic or flat-bottomed restraint after decoupling. Once only a single distance restraint is active, the free energy of release can be calculated exactly, as is done for the DBC restraint.^28,29^ The free energy of releasing a single distance restraint can be calculated using
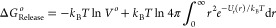
11where *r* is the distance between two anchor atoms, and *U*_r_(*r*) takes the form of eq 8 or 9.

## 3 Methods

### 3.1 System Preparation

The MIF/MIF180 systems were set up approximately following the methodology of Qian et al.^34^ The structures of MIF and MIF180 were obtained from the crystal structures of complexes of MIF with both MIF180 (relatively poor resolution −2.6 Å, PDB ID 4WR8) and the structurally similar MIF190 (relatively high resolution −1.8 Å, PDB ID 4WRB).^45^ Five extra copies of the biological assembly were removed from 4WR8 by deleting all chains from D onward. The higher resolution structure was superimposed on the lower resolution structure by aligning chain B from 4WR8 with chain A from 4WRB using PyMOL.^35^ Crystallographic waters (from 4WRB) were retained, and all other nonprotein and nonligand atoms were discarded. For atoms with alternative locations, the location with the greatest occupancy was selected. Thirteen missing atoms were added using pdb4amber.^46^ H++ (version 3.2) and PROPKA (version 3.4.0) were used to suggest the protonation state of all residues.^47−49^ Consistent with the literature for the apo protein,^50^ a large p*K*_a_ shift was predicted in both cases for the N-terminal proline residue which is present in the binding site, which produces the neutral form at a pH of 7. The suggested protonation sites for all histidines were taken from H++. Hydrogens were added to MIF using H++ and a hydrogen ion was removed from the N of each of the protonated terminal prolines so as to maintain the hydrogen bond to Tyr36. Based on the results of Qian et al., the neutrality of the terminal proline was maintained in the complex.

There are no parameters available for neutral N-terminal proline in the AMBER ff14SB force field.^51^ Therefore, antechamber 21.0 was used to parametrize the neutral proline with an NME-capped C-terminus, using AM1-BCC partial charges and AMBER atom types.^52,53^ The remainder of the MIF protein was parametrized using the AMBER ff14SB force field. Hydrogens were added to MIF180 using Open Babel (version 3.0.0), and BioSimSpace (version 2020.1.0) was used to parametrize the ligand in the *syn* conformation (Figure S2) with the GAFF2.11 force field and AM1-BCC charges using antechamber 21.0.^54,55^

#### 3.1.1 Absolute Binding Free Energy Calculations

Alchemical ABFE calculations were performed using the double decoupling method,^38^ according to the cycle shown in Figure 2 and the multiple distance restraint schemes shown in Figure 5:
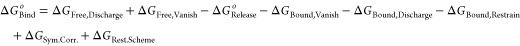
12
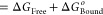
13
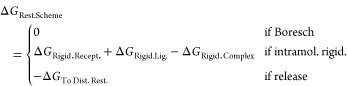
14where *ΔG*_Rest. Scheme_ collects any additional terms which are specific to the restraints scheme used. The relevant restraints scheme for each form of *ΔG*_Rest. Scheme_ is shown on the right of eq 14: “Boresch” includes both Boresch restraints and the “non-rigorous” implementation of multiple distance restraints shown in Figure 5, “intramol. rigid.” refers to multiple distance restraints with intramolecular rigidification, and “release” denotes multiple distance restraints with release to a single distance restraint. *ΔG*_Rigid. Recept._ and *ΔG*_Rigid. Lig._ are the free energy changes for intramolecular rigidification of receptor and ligand anchor points, −*ΔG*_Rigid. Complex_ accounts for the release of intermolecular restraints in the receptor-interacting ligand complex, and *ΔG*_To Dist. Rest._ is the free energy of releasing several distance restraints to a single distance restraint. For the intramolecular rigidification scheme, the intramolecular restraints are applied in State 3, and released in State 7. *ΔG*_Sym. Corr._ is at least – *k*_B_*T* ln 3 for this system, because MIF is a homotrimer with three equivalent binding sites, and all restraints restrict the ligand to a single binding site. *ΔG*_Sym. Corr._ may be larger if the restraints also break symmetries arising from the structure of the ligand (Section S6). *ΔG*_Free_ and *ΔG*_Bound_^*o*^ are the overall free and bound leg contributions to *ΔG*_Bind_^*o*^, where *ΔG*_Bound_^*o*^ includes all terms other than *ΔG*_Free, Discharge_ and *ΔG*_Free, Vanish_.

No correction is included to account for the truncation of the tails of the LJ potentials because initial simulations yielded negligible corrections (≈ 0.1 kcal mol^–1^).^56^ Protein–ligand restraints were introduced, and charges and LJ interactions were incrementally removed by scaling the coupling parameter, λ, from 0 to 1. For calculations with Boresch restraints and multiple distance restraints without intramolecular rigidification or release to a single distance restraint, the force constants of the protein–ligand restraints and the magnitude of the charges were scaled linearly with λ. The soft-core potential implemented in Sire (with a LJ soft-core parameter set to 2.0), which is based on the potentials of Zacharias et al.^57^ and Michel et al.,^58^ was used to scale the LJ interactions. Eight evenly spaced windows and 18 nonevenly spaced λ windows (0.000, 0.028, 0.056, 0.111, 0.167, 0.222, 0.278, 0.333, 0.389, 0.444, 0.500, 0.556, 0.611, 0.667, 0.722, 0.778, 0.889, and 1.000) were used for the free discharging and vanishing stages, respectively. Six nonevenly spaced windows (0.000, 0.125, 0.250, 0.375, 0.500, and 1.000), eight evenly spaced windows, and 36 nonevenly spaced windows (31 evenly spaced windows from λ = 0 to 0.750, then five evenly spaced windows from λ = 0.750 to 1.000) were used for the bound restraining, discharging, and vanishing stages, respectively. The window spacings were selected to yield sufficient overlap without excessive numbers of windows based on initial test simulations. The intramolecular components of both the Coulombic and LJ interactions between ligand atoms were completely removed.

For calculations where multiple distance restraints were released to a single distance restraint, *ΔG*_Release_^*o*^ was calculated by numerical integration of eq 11, and the force constants were scaled with λ^5^ over 21 evenly spaced λ windows.^29^ The same protocol was used to introduce and remove all restraints in the multiple distance restraints simulations with intramolecular restraints.

Restraints were selected to optimally mimic native protein–ligand interactions by postprocessing a 6 ns simulation of the fully interacting complex.^23,26^ From the first frame, all heavy atoms in the protein within 10 Å of the ligand, and all heavy atoms in the ligand were selected. To avoid anchor points with poor correlation in position, the distances between all possible protein–ligand atom pairs from this selection were tracked over the trajectory, and the 200 pairs with the lowest standard deviation were selected. For multiple distance restraints, only the lowest variance pair for any anchor point was retained, provided that neither of the anchor points had already been selected for use in another restraint. For Boresch restraints, all pairs were taken as candidate anchor points a and A (Figure 3). For each pair, adjacent heavy atoms were selected to complete the sets of Boresch anchor points. These sets were ordered by increasing total variance of the Boresch DoF, as done by Alibay,^40,41^ and sets of anchor points were discarded if the average values of θ_A_ or θ_B_ were below 30 or above 150 degrees. The equilibrium values for all restraints were taken to be their average values during the unrestrained simulation. Force constants were selected so that in the decoupled state, the harmonic restraints would generate the same distributions as observed in the coupled state.^23^ Gaussian distributions in the coupled state were assumed and the variances of the Boresch DoF were used to calculate the force constants (Section S7).

For the Boresch restraints, *ΔG*_Release_^*o*^ was calculated by numerical integration of eq 6. This was used in preference to the analytical correction to avoid potential errors introduced by the approximations required to derive eq 7. For multiple distance restraints, numerical integration of eq 10 was performed using the “standardstatecorrection” utility available within Sire,^59^ using all frames of the trajectory, a buffer of 5 Å, a translational volume element of 0.25 Å, and 30 orientations per [0, 2π] Euler angle interval.

#### 3.1.2 Molecular Dynamics Protocol

Solvation and equilibration were performed using BioSimSpace.^55^ The protein–ligand complex was placed in a periodic cube of side 84 Å (determined by the longest edge of the axis-aligned bounding box plus 15 Å, padding on each side) and solvated with TIP3P water molecules.^60^ Then, 150 mM NaCl was added. The system was energy minimized using PMEMD (50,000 steps).^46^ Equilibration in the NVT ensemble was performed using PMEMD (5 ps with all nonsolvent atoms restrained and heating from 0 to 298 K, followed by 50 ps with only backbone atoms restrained, then 50 ps with no restraints), followed by equilibration in the NPT ensemble at 1 atm and 298 K using PMEMD.CUDA (400 ps with all nonsolvent heavy atoms restrained, followed by 2 ns with no restraints).

The free ligand was solvated with TIP3P water and 150 mM NaCl in a periodic box of side length 40 Å. Then, 50,000 steps of minimization were performed using PMEMD. Equilibration in the NVT ensemble was performed using PMEMD (5 ps with all nonsolvent atoms restrained and heating from 0 to 298 K, followed by 50 ps with no restraints), followed by equilibration in the NPT ensemble with PMEMD.CUDA (1 atm and 298 K with restraints on nonsolvent heavy atoms for 200 ps followed by 2 ns with no restraints). A Langevin thermostat and Berendsen barostat were used for the relevant equilibration steps.^61^

All alchemical simulations were performed using the software SOMD,^62^ available within Sire (version 2022.2.0).^59^ SOMD was modified to allow the use of Boresch restraints, the scaling of restraints with λ^5^, and the simultaneous use of different restraints. The code implementing Boresch restraints has been integrated into the main branch of Sire. An Andersen thermostat (collision frequency 10 ps^–1^) and Monte Carlo barostat (25 time steps between isotropic box scaling attempts) were used to maintain a temperature and pressure of 298 K and 1 atm.^63,64^ A time step of 4 fs was used in combination with the leapfrog Verlet integrator and hydrogen mass repartitioning (using a repartitioning factor of 4).^65^ All bond lengths were constrained. The reaction field method was used with a dielectric constant of 78.3,^66^ and a cutoff of 12 Å was used for all nonbonded interactions. Energy minimization was performed prior to each simulation with a maximum of 1000 iterations. The bound stage vanish λ windows were run for 8 ns, and all others for 6 ns. Free energy differences for each stage were estimated using the Multistate Bennett-Acceptance Ratio (MBAR) for the final 5 ns of all simulations.^67,68^ Coordinates were saved every 20 ps.

All simulations were repeated five times with independent starting velocities. Because the MBAR uncertainties estimated from single runs were small compared to the variation between repeat runs, errors are reported as 95% confidence intervals based on the deviation between independent replicates, assuming Gaussian distributions and using *t*-values for 4 degrees of freedom. Student’s *t*-test was used to assess evidence for a significant difference at 95% confidence.

## 4 Results and Discussion

### 4.1 Boresch Restraints

#### 4.1.1 Restraint Selection

**Figure 6:**
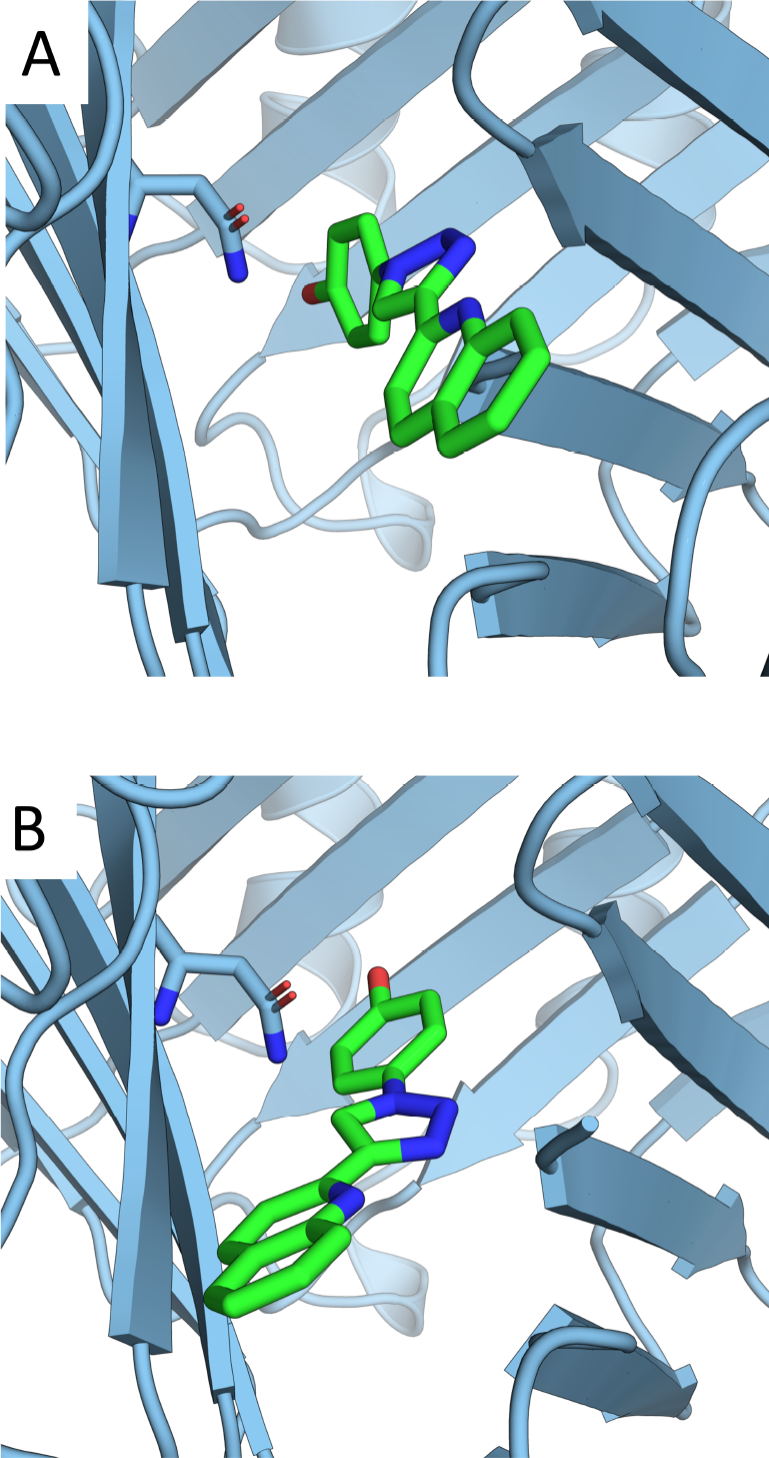
Alternative binding poses A (panel A) and B (panel B). Interconversion occurred rarely on the time scale of the simulation (6 ns). The Asn on which restraints B1 and B1 poseB are based is shown. Rendered with PyMOL.^35^

From an initial set of restraining simulations, two binding poses were identified (Figure 6) which interconverted slowly on the time scale of the simulations (6 ns). To allow comparison of different restraints for a single binding pose, all restraints were fit to binding pose A, other than a single set of Boresch restraints which was fit to pose B. The calculation for pose B was carried out to allow comparison to the experimental free energy of binding.

**Figure 7:**
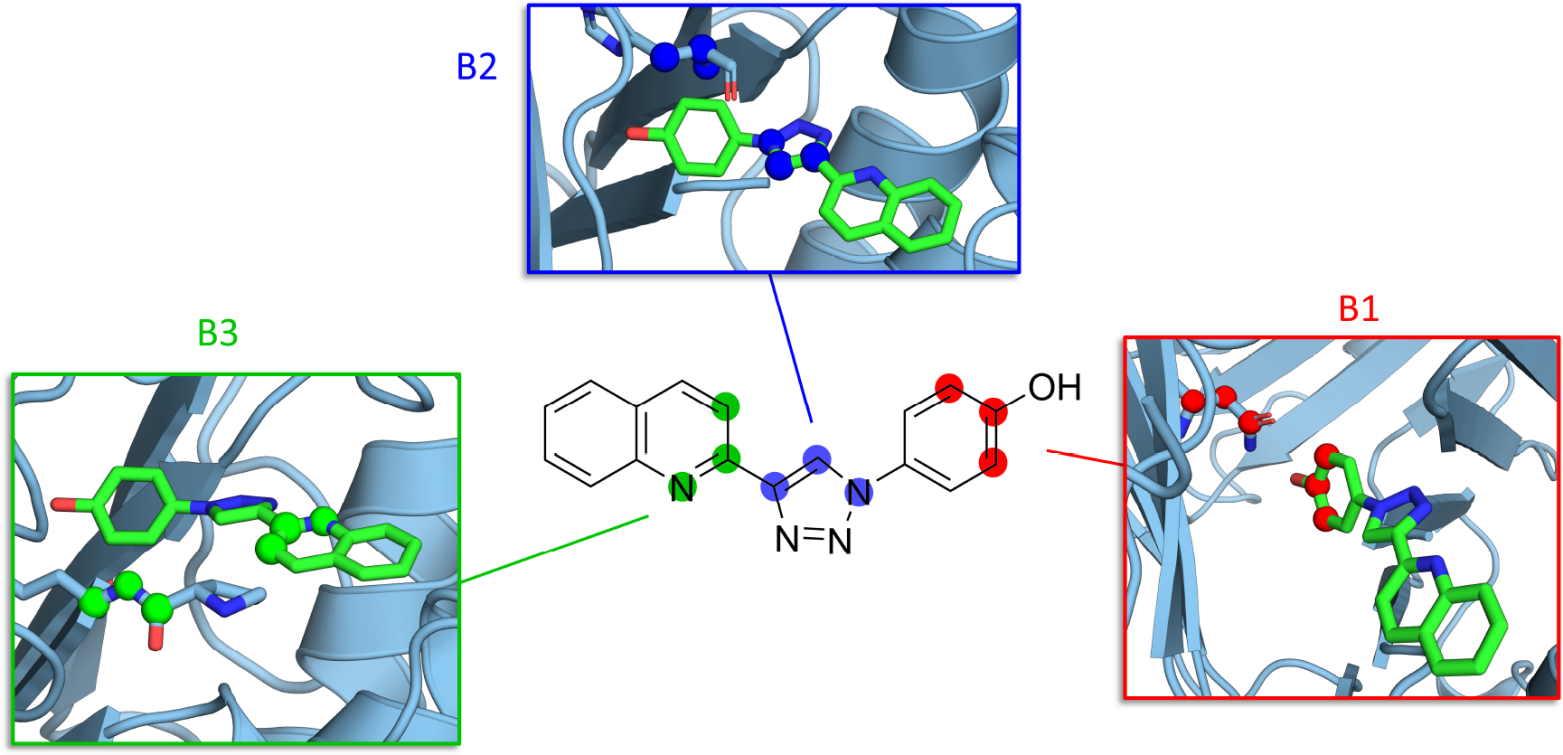
Sets of Boresch anchor points B1 (red), B2 (blue), and B3 (green). Anchor points are circled or shown as spheres. Windows rendered with PyMOL.^35^

Three sets of Boresch restraints were selected initially for pose A (Figure 7). The first was the best-scoring set (B1) based on the minimum-variance algorithm, and mimics the phenol-Asn97C hydrogen bond (Figure 7). To test varied anchor point positions, the second set (B2) was selected as the top-scoring restraints with anchor points outwith the phenol moiety; these were based on the triazole ring and were seventh best-scoring overall. Finally, selection was constrained to the quinoline moiety (B3). The parameters of restraint sets B1, B2, and B3 are given in Table S1. The protein anchor points were located in residues with low root-mean-square fluctuations (RMSFs) of the α-carbon positions although this was not directly targeted by the algorithm (Figure S3). B1-poseB was the highest scoring set of anchor points based on a simulation including only pose B. Similar to the B1 restraints, these were based on the phenol group of MIF-180 and Asn97C.

A discussion of the challenges faced during restraint selection, symmetry corrections, and the strengths and limitations of the restraint selection algorithm is given in Section S6. Improvements to the restraints selection algorithm are also proposed; in particular, we recommend scoring possible restraints using a metric calculated from the variances of the DoF of the prospective restraints, rather than using the total variances directly.

#### 4.1.2 Results with Force Constants Fit to Simulation

Calculations were performed with Boresch restraints with force constants fit to simulation. Force constants were fit based on the variance of the Boresch DoF in State 7, as discussed in Section S7. Several calculations were performed for pose A (B1, B2, and B3) to allow comparison between restraints (Table 1). A single calculation was performed for pose B (B1-poseB) to allow overall comparison with experiment by combining the results for both poses.

**Table 1:** Bound Leg Contributions to *ΔG*_Bind_^*o*^ with Boresch Restraints^a^

	**Restraints**								
**Contribution**	**B1**	**B2**	**B3**	**B1-P**	**B3-P**^b^	**B1-10**	**B2-10**	**B3-10**^c^	**B1-20**
–*ΔG*_Release_^*o*^	9.76	9.90	9.10	10.02	8.91	6.69	6.39	6.53	7.90
–*ΔG*_Bound, Vanish_	–2.06 ± 1.08	–2.20 ± 0.94	–0.73 ± 0.25	–2.22 ± 0.19	–0.68 ± 0.06	–1.04 ± 1.21	0.09 ± 1.03	0.58 ± 1.96	–1.98 ± 1.18
–*ΔG*_Bound, Discharge_	–12.99 ± 0.63	–11.90 ± 0.48	–11.86 ± 0.22	–11.76 ± 0.19	–11.71 ± 0.77	–12.46 ± 0.59	–11.62 ± 0.47	–12.04 ± 0.70	–12.64 ± 0.19
–*ΔG*_Bound, Restrain_	–1.48 ± 0.04	–1.74 ± 0.03	–1.70 ± 0.11	–1.66 ± 0.08	–1.77 ± 0.11	–0.40 ± 0.01	–0.59 ± 0.08	–0.94 ± 0.21	–0.77 ± 0.20
*ΔG*_Sym. Corr._	–1.06	–0.65	–0.65	–1.06	–0.65	–1.06	–0.65	–0.65	–1.06
*ΔG*_Bound_^*o*^	–7.83 ± 1.25	–6.60 ± 1.05	–5.84 ± 0.36	–6.69 ± 0.28	–5.91 ± 0.78	–8.27 ± 1.35	–6.37 ± 1.13	–6.53 ± 2.09	–8.55 ± 1.21

a Results for binding pose A, in kcal mol^–1^. Uncertainties stated as 95% confidence intervals based on the variance of five replicate runs, assuming Gaussian distributions. Restraint parameters are given in Table S1; -10 and -20 denote that all force constants were set to 10 or 20 kcal mol^–1^ Å^–2^ [rad^–2^], respectively.

b Run 1 excluded from average due to undersampling of water in binding site during the vanishing stage.

c Due to simulations crashing, results based on four independent replicates with partial completion of many lambda windows.

The results shown for −*ΔG*_Release_^*o*^ were calculated by numerical integration of eq 6. While use of the Boresch analytical correction introduces large errors in certain regimes (force constants very weak, *r* very short, θ_A_ or θ_B_ close to 0 or π rad), only small deviations between the analytical and numerical corrections, no greater than 0.04 kcal mol^–1^, were found for any of the Boresch restraints. This is in accordance with previous studies.^69^

**Figure 8:**
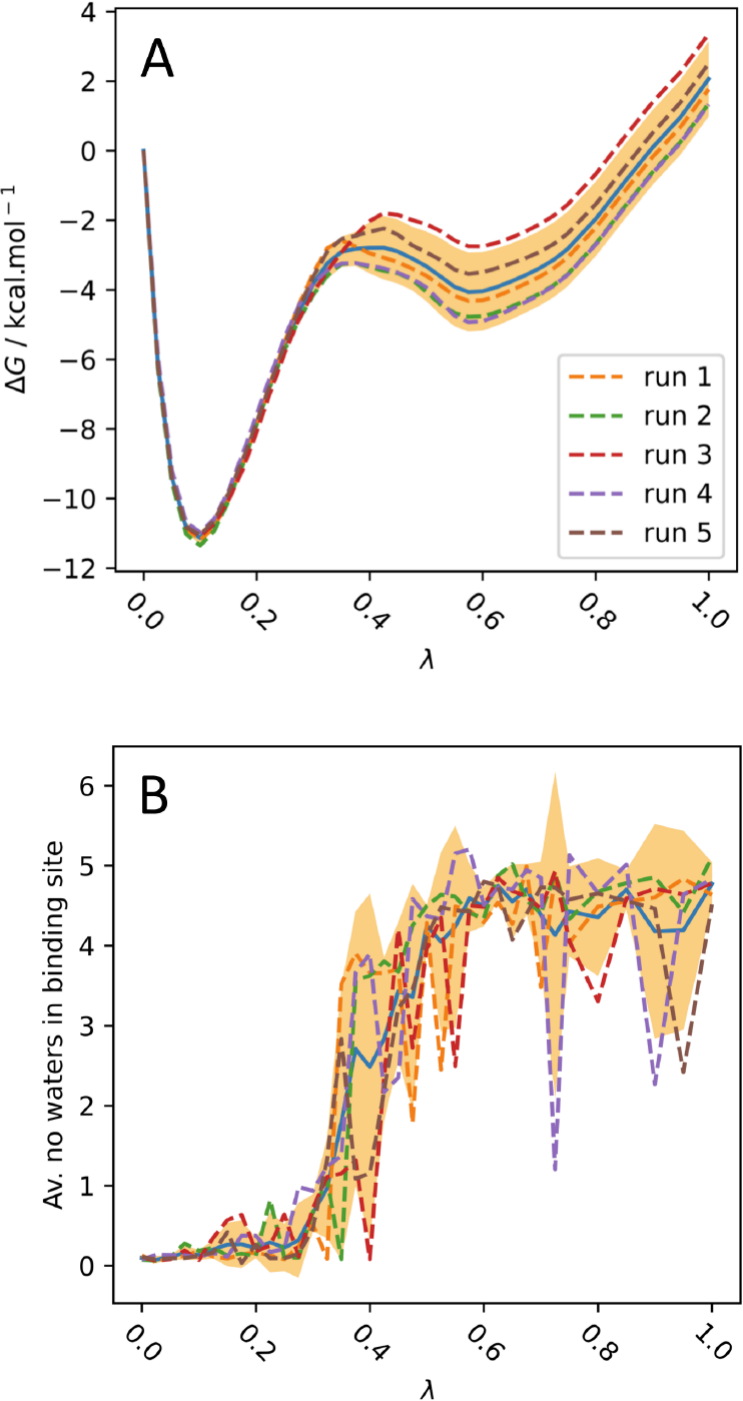
(A) PMF along λ for the bound vanish stage for B1. (B) Average number of waters in the binding site (defined as the overlap of two spheres of radius 8 Å centered on the N atom in Pro1A, and CG2 in Val106A) against λ during the vanish stage for B1. There is a strong correlation between the water occupancy of the binding sites and the divergence of the PMFs around λ = 0.4. The shaded area shows the 95% confidence interval, and the solid blue line shows the mean.

The average of repeat runs for B1-3 and B1-poseB generally showed good convergence as assessed by lack of drift with increasing sampling time (Section S8). However, there were substantial differences between replicate runs, most notably during the vanishing stage, which generally contributed the greatest uncertainty to *ΔG*_Bound_^*o*^. In all cases, this uncertainty could be traced back to a few windows around λ = 0.4 (Figure 8).

**Figure 9:**
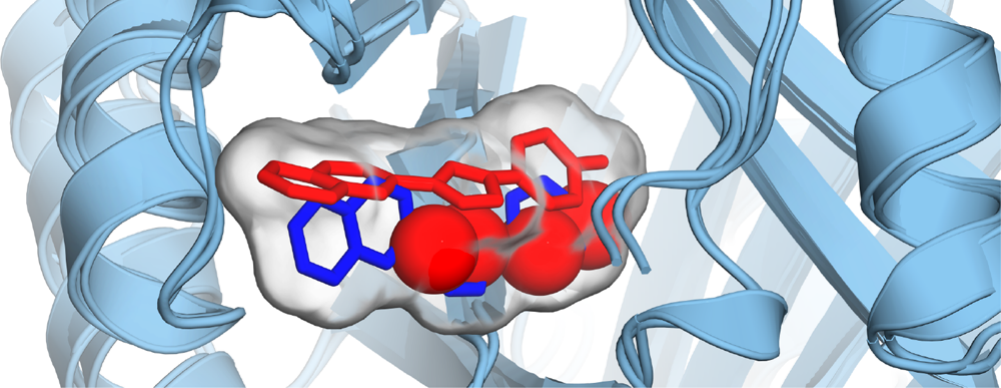
Waters (shown as spheres) in the binding site at λ = 0.4 for B1 at 6.48 ns. Only binding site waters (as defined as the overlap of two spheres of radius 8 Å centered on the N atom in Pro1A and CG2 in Val106A) are shown. The run 1 (red ligand/waters) trajectory was superimposed on that from run 3 (blue ligand/no waters present) by aligning MIF. Surface generated based on the ligands alone to approximately show the binding pocket. The ligand must be pushed to the side of the binding pocket to provide space for the water. Rendered with PyMOL.^35^

The majority of this uncertainty can be attributed to the entry of water to the binding site. Binding site water was defined as being simultaneously within 8 Å of the N atom in Pro1A, and CG2 in Val106A, which are on opposite sides of the binding site. At the start of the vanishing stage there were no waters in the binding site, which increased to an average of approximately 4.5 after decoupling, in good agreement with the crystal structure of free MIF (PDB ID 1GD0).^70^ A sudden jump in water occupancy of the binding site occurred around λ = 0.4, the region of divergence of the PMFs for B1. Here, it was found that water may only enter the binding site by “forcing” the partially vanished ligand to the side of the binding site (Figure 9).

In cases where the binding site showed high water occupancy (runs 1, 2, and 4), the resultant strain favored vanishing of the ligand, producing divergence of the vanish stage PMF toward more favorable free energies of vanishing. The opposite was true for runs 3 and 5, which had very low average water occupancies at λ = 0.4. This is in accord with the results of Rogers et al.,^71^ who found negative correlation between water occupancy and the gradient of the potential energy with respect to λ at intermediate stages of the vanishing leg. Although dramatic fluctuations in the number of water molecules in the binding sites were observed at higher values of λ, this did not translate into additional uncertainty, because at this stage the LJ terms are mostly removed and the ligand is able to pass through atoms relatively smoothly. Some dips in the water occupancy were found to be due to the rotation of the side chain of Met2A to obstruct the end of the binding site.

Based on preliminary simulations, the equilibration time for the vanish stage simulations was increased to 3 ns per window to remove systematic error from slow movement of water into the binding site. However, as shown by Figure 8, occasionally water failed to enter the binding site during the entire simulation at values of λ where several waters entered the binding site during replicate runs. Therefore, some systematic error likely persisted in some cases. This appeared to be true for the B1 simulations. Irrespective of whether the force constants were fit to simulation (B1), or set to 10 (B1-10) or 20 (B1-20) kcal mol^–1^ Å^–2^ or kcal mol^–1^ rad^–2^, the results were more negative than for B2 and B3. In most cases the differences were significant at 95% confidence. In addition, these simulations generally showed the greatest uncertainties between replicates, demonstrating greater random error. This was despite the B1 restraints being selected as optimum based on the minimum variance algorithm, and the protein anchor points being based on a stable Asn forming part of a β-sheet, highlighting the difficulty of selecting optimal restraints.

It seemed unlikely that the offset was due to the restraint of the phenol group, because multiple distance restraint schemes based on this group did not produce such negative free energies of vanishing (see Section 2.4). To ensure that this was not due to flexibility of the Asn side chain, the calculation was repeated using ligand anchors in the phenol but protein anchors only in the backbone and Cα of this Asn. The result was very similar (−7.88 ± 0.95 kcal mol^–1^), showing that the issue was not side-chain flexibility. Finally, the calculation was repeated using ligand atoms in the phenol but protein anchors in a different residue (Ile64A), see B1-P. This resulted in a *ΔG*_Bound_^*o*^ of −6.69 ± 0.28 kcal mol^–1^ which had a much smaller uncertainty and was closer to the results for B2 and B3. The difference between B1 and B1-P was significantly different based on a Student’s *t* test, although it is acknowledged that the assumption of normally distributed free energy differences is likely to be incorrect.^72^ In this system especially, where the dominant source of error appears to be the slow hopping of water between energy minima, the central limit theorem is unlikely to be applicable. Regardless, the B1-P results were substantially less negative than the B1-10 and B1-20 results, suggesting that basing the protein anchors on the Asn for the B1 restraints may have made the simulations more susceptible to water sampling issues. It is possible that the restraints between the phenol and this Asn, which quickly forms a H-bond to water upon its entry to the binding site, created particularly high barriers to the entry of water, giving rise to systematic error. Regardless, the difference observed suggests that the approach of running independent replicate simulations with different restraints, as taken by Alibay et al.,^40^ is sensible.

The B3 simulations were also rerun with protein anchor atoms on a different residue (Ala38A), see B3-P. There was very good agreement between runs 2–5 for the bound vanish stage, but *ΔG*_Bound_^*o*^ was around 3 kcal mol^–1^ more negative for run 1 due to comparatively low number of waters in the binding site over just 3 λ windows (see Section S9). This highlights the importance of correctly sampling rehydration of the binding site upon decoupling.^73^ It has been demonstrated that hybrid sampling approaches combining molecular dynamics with Monte Carlo water moves, in both the μVT and NPT ensembles, can improve the performance of relative binding free energy calculations.^74−76^ In this system, it is possible that these approaches may perform poorly as a result of low acceptance probabilities, because water has to strain the partially decoupled ligand to enter the binding site around λ = 0.4, where proper sampling of rehydration is most critical. Nonequilibirum candidate Monte Carlo may overcome this by allowing for relaxation of the ligand position.^73^ The strong dependence of the free energy on the correct sampling of binding-site water may make this a good system for testing methods for enhancing sampling of rehydration. Regardless, the *ΔG*_Bound_^*o*^ obtained after discarding the data from run 1 (−5.91 ± 0.78 kcal mol^–1^) was very similar to that obtained for B3 (−5.84 ± 0.36 kcal mol^–1^).

Ignoring the results for B1, which seemed to be especially affected by water sampling issues, the remaining simulations with force constants fit to simulation showed generally good agreement (within 1 kcal mol^–1^), demonstrating reasonable reproducibility with this restraints scheme. Averaging B2, B3, B1-P, and B3-P yielded a *ΔG*_Bound_^*o*^ of −6.26 ± 0.72 kcal mol^–1^ for binding pose A.

Although the aim of this study was to compare the values of *ΔG*_Bound_^*o*^ obtained with different restraints, a single set of five replicate calculations were carried out for the free leg to allow comparison with experiment. Comparison with the experiment cannot be used for the comparison of restraint schemes as there are likely to be systematic errors from the force field, but it is used here as a crude check on the overall results. There was excellent agreement between replicates of the free leg simulations, and convergence was achieved quickly (Section S10), yielding *ΔG*_Free_ = −3.08 ± 0.14 kcal mol^–1^ (*ΔG*_Free, Discharge_ = 9.03 ± 0.07 kcal mol^–1^, and *ΔG*_Free, Vanish_ = −12.11 ± 0.11 kcal mol^–1^). This was in spite of *syn–anti* interconversion, which was observed during at least one run for every lambda window, but which occurred slowly on the time scale of the simulations. *ΔG*_Bound_^*o*^ for binding pose B (with the B1-poseB restraints) was −6.63 ± 1.11 kcal mol^–1^ (−*ΔG*_Release_^*o*^ = 9.76 kcal mol^–1^, – *ΔG*_Bound, Discharge_ = −2.66 ± 1.04 kcal mol^–1^, – Δ*G*_Bound, Vanish_ = −10.77 ± 0.39 kcal mol^–1^, – *ΔG*_Bound, Restrain_ = −1.90 ± 0.1 kcal mol^–1^, and *ΔG*_Sym. Corr._ = −1.06 kcal mol^–1^). Combining the average result for pose A (−6.28 ± 0.49 kcal mol^–1^, using all results from Table 1 excluding all B1 calculations and B3-10 due to the issues discussed) with the result for pose B according to *ΔG*_Bound_^*o*^ = −*k*_*B*_*T* ln (exp(−*βΔG*_Bound, 1_^*o*^) + exp(−*βΔG*_Bound, 2_^*o*^)) yielded an overall *ΔG*_Bound_^*o*^ of −6.89 ± 0.74 kcal mol^–1^ and *ΔG*_Bind_^*o*^ = −9.97 ± 0.76 kcal mol^–1^.^37^ The overall result was in good agreement with the experimental binding free energy of −8.98 ± 0.28 kcal mol^–1^,^77^ but more negative than the value calculated by Qian et al. using molecular dynamics and the AMBER ff14SB and GAFF force fields (−7.47 ± 0.99 kcal mol^–1^).^34^ However, a clear comparison with the results of Qian et al. is prevented by a number of methodological differences. For example, they only observe the *anti* and *syn* conformers of MIF180 during the free and bound legs, respectively, and they apply a penalty of 1.60 kcal mol^–1^ to account for this. Here, interconversion was observed in both the free and bound states, and no correction was applied. Furthermore, Qian et al. do not apply a symmetry correction to account for the 3-fold symmetry of MIF, and do not perform calculations for an alternative binding pose.

#### 4.1.3 Results without Orientational Restraints

It was found that orientational restraints were essential for achieving reliable free energy estimates. The requirement for orientational restraints was investigated by repeating the B1 and B2 calculations without the orientational component of the restraint (*K*_θ_B__, *K*_ϕ_B__, and *K*_ϕ_C__ were set to 0), see B1-o and B2-o in Table 2. B1 was also repeated setting all force constants other than *K*_*r*_ to 0, thus retaining only a single distance restraint, see B1-d. This resulted in large and significant shifts to more negative free energies of binding by 1.72, 3.84, and 2.05 kcal mol^–1^, for B1-o, B2-o, and B1-d, respectively. Despite this, there was no obvious drift of the free energies with increasing sampling time (Section S11).

**Table 2:** Bound Leg Contributions to *ΔG*_Bind_^*o*^ without Orientational Restraints^a^

	**Restraints**		
	**B1-o**	**B2-o**	**B1-d**
–*ΔG*_Release_^*o*^	4.94	4.51	1.08
–*ΔG*_Bound, Vanish_	–0.60 ± 0.71	–1.47 ± 0.99	1.67 ± 1.11
–*ΔG*_Bound, Discharge_	–12.50 ± 0.40	–11.93 ± 0.20	–11.89 ± 0.44
–*ΔG*_Bound, Restrain_	–0.74 ± 0.01	–0.91 ± 0.14	–0.09 ± 0.00
*ΔG*_Sym. Corr._	–0.65	–0.65	–0.65
*ΔG*_Bound_^*o*^	–9.55 ± 0.82	–10.44 ± 1.02	–9.88 ± 1.19

a All values in kcal mol^–1^. Uncertainties stated as 95% confidence intervals based on the variance of five replicate runs, assuming Gaussian distributions; -o and -d mean that all force constants other than *k*_*r*_, *k*_θ_*A*__, and *k*_ϕ_*A*__, or *k*_*r*_ were set to 0, respectively.

The lack of orientational restraints allows the mixing of binding poses A and B, but this is also not a plausible source of the error introduced. In the limit of perfect sampling, the free energy of binding can be no more negative than that calculated by combining the free energies of binding of the two poses as was done previously. The exception to this would be if there were other binding poses which were numerous or more favorable, which seems unlikely.

Instead, the negative offset is very likely due to the failure to sample all relevant orientations at intermediate values of λ during vanishing. The close agreement between B1-o and B1-d suggests that the offset is due to the removal of the orientational component of the restraint. As the LJ interactions are removed, the sampling of orientations different to that of the binding pose will become favorable. The gradient of the free energy change as the LJ interactions are removed will likely be less positive in these alternative orientations, because they were high in energy when the LJ interactions were at full strength. Therefore, failure to sample these orientations due to large barriers should give excessively positive free energies of vanishing, resulting in erroneously negative free energies of binding, as is observed.

**Figure 10:**
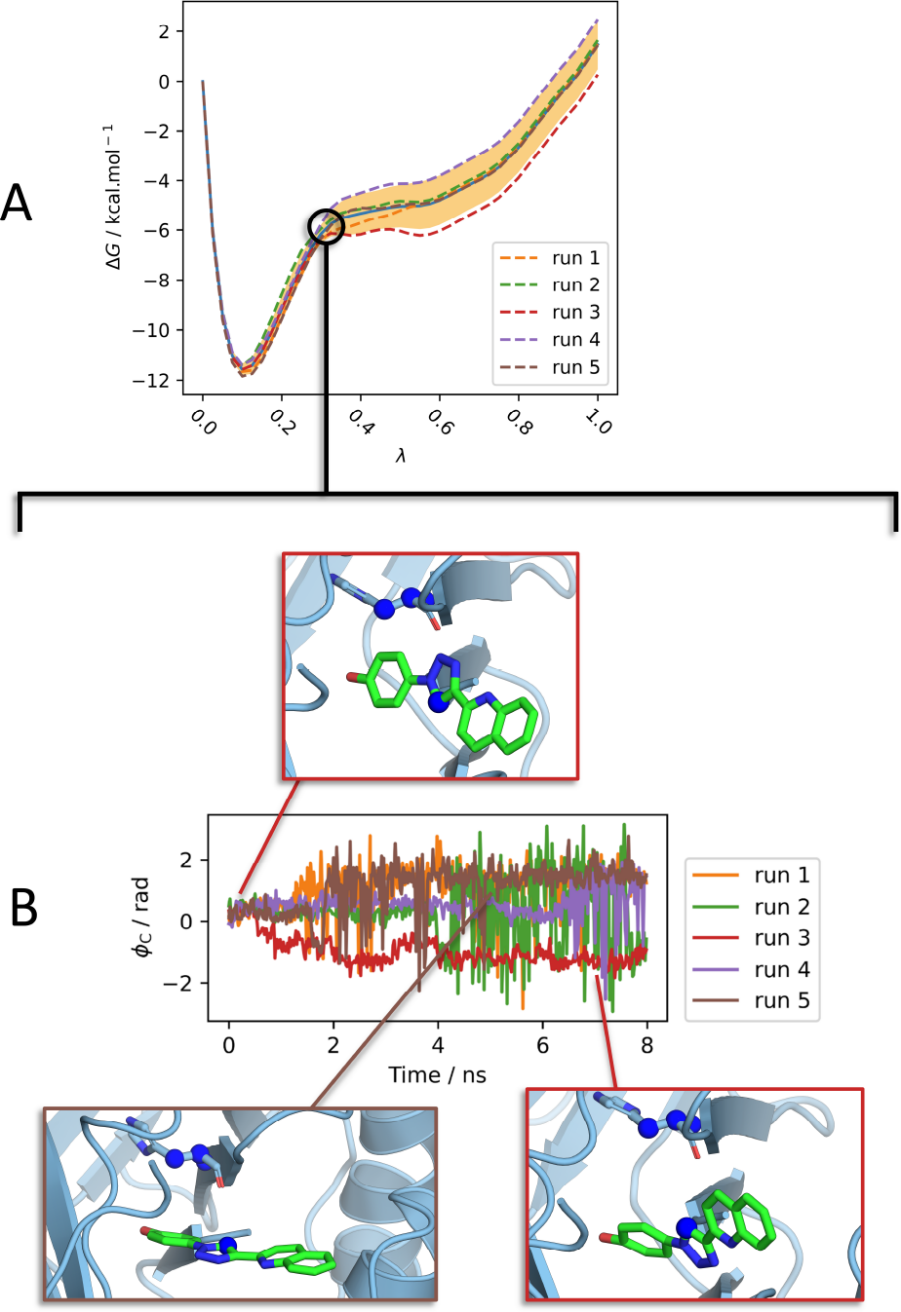
(A) The PMFs for the bound vanish stage for B2-o. These diverge around λ = 0.325. (B) The unrestrained Boresch DoF ϕ_C_ for B2-o at λ = 0.325, showing the presence of multiple slowly interconverting orientations. Snapshots taken from run 3 (0.06 ns), run 5 (5.06 ns), and run 3 (7.22 ns), from left to right. The anchor points used to define ϕ_C_ are shown as spheres. Windows rendered with PyMOL.^35^

This is illustrated by the divergence of the PMF for the bound vanish stage for B2-o (Figure 10). The divergence shows a strong correlation with the orientational sampling at λ = 0.325, and not with the presence of water in the binding site (Figure S16). For run 4, the plots of ϕ_C_ show that sampling was largely restricted to the orientation of the original binding mode. This resulted in the most positive gradient of the PMF and the most favorable free energy of binding. During runs 1, 2, and 5, the ligand rotated lengthwise in the binding site by around 90° with respect to the initial pose, resulting in a less positive PMF gradient. During run 3, the ligand rotated around 180° lengthwise in the binding site with respect to the initial pose. The gradient here was evidently even less positive, resulting in a substantially less negative free energy of binding. The slow interconversion between orientations explains the lack of drift of the results with increasing simulation time; there are large barriers between orientations which prevent equilibrium sampling on the time scale of the simulations. To support this explanation, the free energy of releasing B1 to B1-o in the decoupled state was calculated by scaling the strength of the orientational force constants with λ, using the same set of λ windows as for the bound vanish stage. The free energy difference for releasing the restraints calculated by simulation was 4.92 ± 0.02 kcal mol^–1^ (MBAR 95% C.I. estimate for a single simulation), in close agreement with the difference calculated by numerical integration by subtracting the two *ΔG*_Release_^*o*^ terms (4.82 kcal mol^–1^). This confirms that sampling a large increase in configurational space is not problematic when there are no barriers, at least when the growth is sufficiently slow; the issue is sampling the “rugged” configurational space at intermediate stages of decoupling. Comparison to prior work on the use of orientational restraints is given in Section S13.

The offset introduced by the removal of orientational restraints may be reduced or removed through the use of the Hamiltonian-replica exchange (HREX) method. This is because in the fully decoupled state, there are no barriers to orientational rearrangements, and HREX has been found to improve sampling when the barriers in configurational space are low in at least one state.^78^ This allows free sampling of varied orientations, and mixing of these into the intermediate λ states using HREX may improve orientational sampling. However, it may not remove the bias; Lapelosa et al. found that convergence of their HREX ABFE calculations for a large and flexible ligand could only be achieved with orientational restraints.^79^

#### 4.1.4 Performance of Common Default Force Constants

The performance of the Boresch restraints with the force constants fit to simulation were compared to those with all force constants set to the common defaults of 10 or 20 kcal mol^–1^ Å^–2^ [rad^–2^], denoted by -10 and -20 (Table 1).^37,80,81^ No significant differences in *ΔG*_Bound_^*o*^ were found compared to when the force constants were fit to simulation, which was unsurprising given that the theoretical independence of the binding free energy with respect to the strength of restraints has been previously confirmed.^17,69^ If there were any improvements in precision or increases in the rate of convergence with the force constants fit to simulation, these were not observed above the noise generated by other sources of error.

The only difference when default force constants were used was that several simulations crashed, very likely due to the collinearity of contiguous anchor points. While all simulations completed successfully when the force constants were fit to simulation, 1 λ window failed for B2-10 (this was rerun) and 12 failed for B3-10.

**Figure 11:**
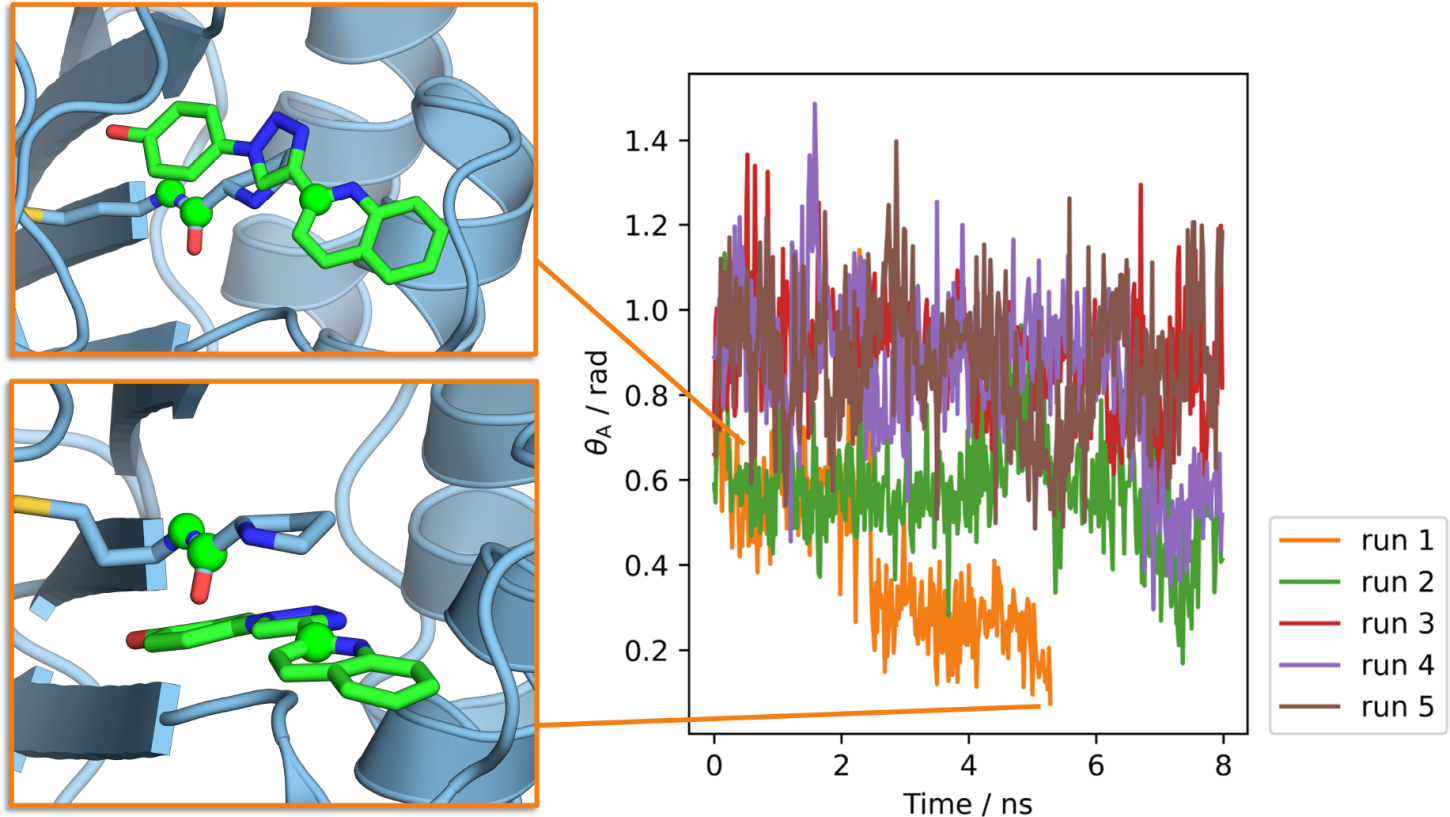
Restrained angle θ_A_ for B3–10 at λ = 0.475 during the vanish stage. For run 1, θ_A_ tended toward 0 as the ligand became trapped under the terminal proline and the simulation crashed. Anchor points used in the definition of θ_A_ are shown as spheres. Snapshots taken from run 1 at 1.12 ns (upper image) and 5.30 ns (lower image). Windows rendered with PyMOL.^35^

For B3-10, the minimum energy penalty arising from the restraints for setting a θ_A_ or θ_B_ to 0 or 180° was approximately 5 *k*_B_*T*, meaning that collinearity was relatively likely and crashes may have been anticipated. However, for B2-10, the minimum penalty for collinearity was approximately 10 *k*_B_*T*. This makes crashes in the decoupled state highly unlikely, but when the ligand is still interacting with the protein it may become trapped in unusual orientations which distort the anchor points toward collinearity. This was the cause of the crash for B3-10 run 1: during the vanish λ = 0.475 window, the simulation failed after θ_A_ approaches 0 (Figure 11). This occurred because the ligand became trapped underneath the terminal proline residue, resulting in θ_A_ approaching collinearity. A better restraint selection algorithm would have discounted restraints if the energy penalty from the restraints for collinearity was below some threshold, rather than only checking the equilibrium angles. Regardless, this highlights that instabilities with Boresch restraints can be an issue even when sensible restraint selections are made. It also illustrates that fitting the force constants to simulation (or at least using higher force constants) can produce more stable restraints.

### 4.2 Multiple Distance Restraints

#### 4.2.1 With Intramolecular Rigidification

Multiple distance restraints provide a framework which is free from the inherent instabilities of Boresch restraints, and which allows more complete restraint of the ligand than Boresch restraints alone. However, their naive application renders the ABFE framework theoretically inexact. To illustrate this, harmonic distance restraints were applied to every heavy atom in the ligand and their lowest variance unique partner heavy atom in the protein (protocol M-All, 22 receptor–ligand distance restraints). As expected, this produced an excessively negative *ΔG*_Bound_^*o*^ estimate, in excess of 3 kcal mol^–1^ more negative than most of the Boresch restraints, indicating that *ΔG*_Preorg._ was large (Table 3). The distance restraint dictionaries used for all protocols are given in Section S14.

**Table 3:** Bound Leg Contributions to *ΔG*_Bind_^*o*^ with Multiple Distance Restraints^a^

	**Restraints**						
**Contribution**	**M-All**	**M-Rig**	**M-Rig-N**^b^	**M-All-R**	**M-Hand-R**	**M-Hand**	**M-Hand-1**
*ΔG*_Rigid. Lig._	–	0.50 ± 0.00	–	–	–	–	–
*ΔG*_Rigid. Recept._	–	10.36 ± 0.09	–	–	–	–	–
–*ΔG*_Release_^*o*^	15.68 ± 0.37	10.05 ± 0.17	9.97 ± 0.03	2.40	1.44	4.35 ± 0.27	5.82 ± 0.26
–*ΔG*_To Dist. Rest._	–	–	–	16.66 ± 0.29	3.07 ± 0.24	–	–
–*ΔG*_Bound, Vanish_	–7.71 ± 1.05	–2.48 ± 1.25	–2.83 ± 0.90	–7.71 ± 1.05	1.58 ± 1.05	1.58 ± 1.05	1.81 ± 1.06
–*ΔG*_Bound, Discharge_	–13.54 ± 0.45	–13.23 ± 0.46	–12.97 ± 0.10	–13.54 ± 0.45	–11.89 ± 0.43	–11.89 ± 0.43	–12.99 ± 0.64
–*ΔG*_Bound, Restrain_	–3.67 ± 0.10	–1.33 ± 0.03	–1.35 ± 0.03	–3.67 ± 0.10	–0.02 ± 0.02	–0.02 ± 0.02	–0.28 ± 0.12
–*ΔG*_Rigid. Complex_	–	–8.31 ± 0.08	–	–	–	–	–
*ΔG*_Sym. Corr._	–1.06	–1.06	–1.06	–0.65	–0.65	–0.65	–0.65
*ΔG*_Bound_^*o*^	–10.31 ± 1.20	–5.50 ± 1.35	–8.24 ± 0.91	–6.51 ± 1.18	–6.47 ± 1.16	–6.64 ± 1.16	–6.30 ± 1.27

a Results for binding pose A, in kcal mol^–1^. Uncertainties stated as 95% confidence intervals based on the variance of five replicate runs assuming Gaussian distributions. The distance restraint dictionaries used for all protocols are given in Section S14; -N indicates that the protocol was repeated with no intramolecular rigidification, -R repetition with release to the single strongest distance restraint, and -1 repetition with the flat-bottomed diameter set to 1 Å for all restraints. *ΔG*_To Dist. Rest._ is the free energy of releasing the multiple distance restraints to a single distance restraint.

b 10 replicate runs were used. The convergence of −*ΔG*_Release_^*o*^ with respect to the parameters of the Sire standard state correction utility was confirmed (Section S15).

**Figure 12:**
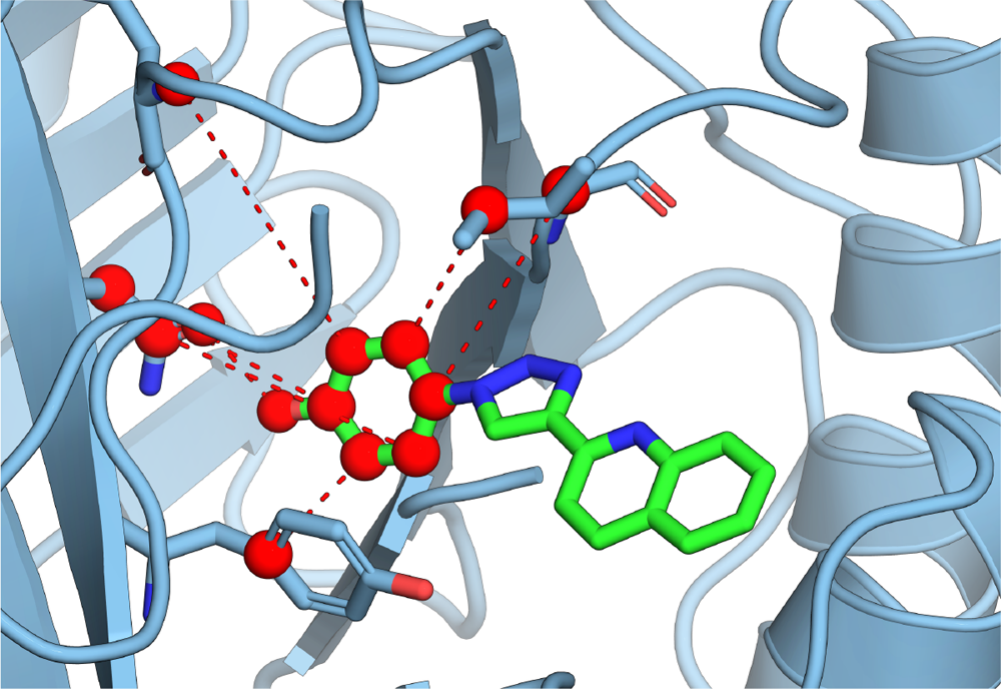
Anchor points (red) used for the M-Rig restraints. Intermolecular restraints are shown as red dashed lines, while intramolecular restraints not shown. Rendered with PyMOL.^35^

This was contrasted with the rigorous multiple distance restraints scheme with intramolecular rigidification of anchor points (Figure 12), referred to as M-Rig. Anchor points were selected as for M-All, except that only anchor points in the phenol moiety of the ligand were used in order to avoid the tight restraints restricting rotatable bonds in the ligand; this would likely require an enhanced sampling approach, such as umbrella sampling,^82^ to restrain bond rotation before applying the restrictive intermolecular restraints. This resulted in seven receptor–ligand distance restraints. The intramolecular restraints were implemented as harmonic distance restraints between all pairs of anchor atoms within the given molecule, with force constants of 75 kcal mol^–1^ Å^–2^. For simulations where −*ΔG*_Release_^*o*^ was calculated using eq 10, it was confirmed that the estimate had converged with respect to the number of points used for numerical integration (Section S15). This scheme gave *ΔG*_Bound_^*o*^ = −5.50 ± 1.35 kcal mol ^–1^, in good agreement with the previous Boresch calculations, providing proof-of-concept of a rigorous implementation of multiple distance restraints.

The magnitude of *ΔG*_Preorg._ can be roughly estimated as *ΔG*_Rigid. Recept._ + *ΔG*_Rigid. Lig._ – *ΔG*_Rigid. Complex_ = 2.55 ± 0.17 kcal mol^–1^ (see Section S18). This is in excellent agreement with the difference observed when the procedure was repeated without intramolecular restraints (M-Rig-N), which was 2.74 ± 1.63 kcal mol^–1^, providing evidence that this is the main source of the error observed when multiple distance restraints are applied in a nonrigorous manner.

The intramolecular restraints are required to prevent distortion of the intramolecular degrees of freedom by the intermolecular restraints, which would otherwise introduce error into the *ΔG*_Release_^*o*^ calculated using eq 10. The strength of the intramolecular restraints required to eliminate this error is dependent on how permissive the intermolecular restraints are: highly restrictive intermolecular restraints necessitate aggressive rigidification.

For the M-Rig calculations, strong intermolecular restraints were used, and therefore, strong intramolecular restraints were also required. The large value of *ΔG*_Preorg._ which resulted from the strong intermolecular restraints allowed us to show that that intramolecular rigidification eliminates this error, by comparison of M-Rig and M-Rig-N. However, the strong intermolecular restraints resulted in a significant additional computational cost during the rigidification stage. The λ protocols for the rigidification stages could likely have been optimized to reduce the total number of windows to approximately 25 (Section S17), but this would still result in an additional computational cost comparable to the vanishing stage. Hence, we would not recommend multiple distance restraints with intramolecular rigidification when using strong intermolecular restraints. Instead, this scheme is expected to perform best with more permissive intermolecular restraints. An efficient implementation may be as follows:

(1) Select flat-bottomed, rather than harmonic, intermolecular restraints and select the flat-bottomed regions so to include almost all distances sampled during the unrestrained simulation of the fully interacting complex.
(2) Perform the rigidification simulations simultaneously starting from States 3 and 4. Monitor the convergence of *ΔG*_Preorg._ as the intramolecular restraint strength is increased and stop the calculations once convergence is achieved.
(3) Calculate *ΔG*_Release_^*o*^ with eq 10 based on the trajectory for the most strongly rigidified version of State 4.

In cases where such flat-bottomed restraints are used, and there is little rearrangement of the ligand or binding site upon decoupling, *ΔG*_Preorg._ would be expected to converge immediately and the above scheme would reduce to the naive multiple distance restraints scheme. When this is not the case, the naive scheme would be expected to yield incorrect results, while the above scheme should remain correct.

#### 4.2.2 With Release to Single Distance Restraint

Based on the approach taken with DBC restraints,^28,29^ the free energy of releasing all but the strongest distance restraint after decoupling was calculated for M-All (M-All-R). Because a single distance restraint does not couple the internal and external degrees of freedom of the protein and ligand, this allows the rigorous calculation of −*ΔG*_Release_^*o*^ using eq 11, removing the requirement for intramolecular restraints.

In contrast to M-All, M-All-R produced a *ΔG*_Bound_^*o*^ of −6.51 ± 1.18 kcal mol^–1^, in good agreement with the Boresch calculations. There appeared to be a slight drift in *ΔG*_To Dist. Rest._ with time (Figure S20) toward more negative values, which may be due to the requirement for the ligand to sample all points on the surface of a sphere surrounding the protein anchor point upon decoupling. This may be improved by releasing to a center-of-mass restraint centered on the binding site, reducing the volume which must be sampled.^29^ In addition, scaling the restraint potential differently between the end points may improve convergence; instead of the λ^5^ scaling used here, a soft bond stretch potential would likely perform well for the removal of these harmonic restraints.^83^

This process was repeated using a significantly more permissive multiple distance restraint scheme, where four flat-bottomed distance restraints were selected to mimic the four protein–ligand hydrogen bonds shown in Figure 1. This scheme is denoted M-Hand-R as the anchor points were selected by hand, although automated selection to match hydrogen bonds would be straightforward. The radius of the flat-bottomed region was selected to be as small as possible without the restraints engaging at any point during the restraint fitting simulation, ensuring that *ΔG*_Bound, Restrain_ ≈ 0. The force constants for the half-harmonic potentials were 40 kcal mol^–1^. This again produced a *ΔG*_Bound_^*o*^ in good agreement with the Boresch results and M-Rig (−6.47 ± 1.16 kcal mol^–1^), indicating that the relatively permissive restraints sufficiently restricted orientational sampling. Furthermore, no substantial drift in the estimate with simulation time was observed (Figure S21).

Compared to the naive and Boresch schemes, this scheme required a single additional release stage, which was relatively computationally affordable. Furthermore, computational cost could be further reduced with optimization of the λ schedule for M-Hand-R, because excellent overlap was achieved between many nonconsecutive λ windows (Section S17). The overlap matrices for the vanishing stages were very similar regardless of the restraint scheme, and the number of windows required for the vanishing stages could not have been reduced below approximately 30 in any case (see Figure S24 as a representative example). In contrast, for both M-Hand-R and M-Rig-R, it appears that 10 windows would be sufficient, or even fewer in the case of M-Hand-R. For M-Hand-R, convergence appears to be achieved after around 1 ns sampling per window (not including the 1 ns equilibration), which seems broadly similar to the bound vanish stage results (not including the 3 ns equilibration). Therefore, when the intermolecular restraints are not extremely numerous and strong, the additional cost associated with the release stage with an optimized λ schedule is expected to be substantially less than a third of the vanish stage.

Although there was no evidence for improved convergence of the decoupling simulations with M-All-R over the Boresch schemes, this might be observed in other systems with highly flexible ligands, where ligand conformational sampling is the dominant source of uncertainty. However, the use of Boresch restraints in combination with RMSD-based restraints on the conformation of the ligand may prove similarly effective.^82^

This scheme is in some ways similar to the DBC scheme, in that a complex restraint (set of restraints) involving many degrees of freedom is released to a single harmonic restraint to allow the calculation of *ΔG*_Release_^*o*^. While the DBC restraint is attractively simple—it consists of a single flat-bottomed restraint on the RMSD of a subset of ligand coordinates in the frame of reference of the binding site—multiple distance restraints schemes offer finer control over the strength of restraints applied to different subsections of the system. This may be beneficial, for example, in the case of a large and flexible ligand where only part of the ligand interacts strongly with the receptor. If the coordinates of all ligand heavy atoms were included in the DBC restraint, then the DBC coordinate would show very wide fluctuations during simulations of the bound state. Fitting the DBC restraint to encompass the 95th percentile of sampled DBC coordinates would result in a very weak restraint on all sections of the ligand, which may result in sampling issues. Flat-bottomed multiple distance restraints could be fit in a similar way, such that the flat-bottomed regions encompassed almost all distances measured during a simulation of the fully interacting complex. In contrast to the DBC restraints fit to all ligand heavy atoms, the multiple distance restraints fit to all heavy atoms would closely restrict the portion of the ligand which interacts strongly with the receptor, while allowing large fluctuations in the flexible portion which does not. While multiple distance restraints require many more parameters than the DBC restraint, they can be automatically selected from a simulation of the fully interacting complex using algorithms described in this work. Furthermore, if the user opted not to run such a simulation for restraint selection, distance restraints could be intuitively selected to match receptor–ligand interactions, and reasonable default parameters could be chosen. It may be challenging to select a reasonable flat-bottomed region for a DBC restraint without a trajectory.

Averaging over the rigorous multiple distance restraint schemes (M-Rig, M-All-R, and M-Hand-R), the mean *ΔG*_Bound_^*o*^ result for pose A was −6.16 ± 1.43 kcal mol^–1^. This was in good agreement with the mean result for pose A calculated with Boresch restraints (−6.28 ± 0.49 kcal mol^–1^, average of B2, B3, B1-P, and B3-P, and B2-10). Ignoring the contribution to the free energy of binding from pose B, for which no calculations were performed with multiple distance restraints, the free energies of binding would have been −9.24 ± 1.44 and −9.36 ± 0.37 kcal mol^–1^ for multiple distance restraints and Boresch restraints, respectively.

#### 4.2.3 With a Large Flat-Bottomed Region

Finally, a nonrigorous implementation of multiple distance restraints was tested, based on the assumption of no coupling between internal and external degrees of freedom in the limit of weak restraints. The schemes tested were M-Hand, a repetition of M-Hand-R without releasing to a single distance restraint, and M-Hand-1, a repetition of M-Hand with all flat-bottomed diameters set to 1 Å, a reduction from the average diameter of 2.7 Å for M-Hand-R.

A *ΔG*_Bound_^*o*^ of −6.64 ± 1.16 kcal mol ^–1^ was obtained for M-Hand. This was not significantly different to M-Hand-R or M-Rig, suggesting that the approximations made by the scheme were minor in this case. The results for M-Hand and M-Hand-1 (−6.30 ± 1.27 kcal mol^–1^) were very similar, despite the slightly more restrictive restraints. This shows that it is possible to obtain equivalent free energies using the naive distance restraints scheme and rigorous schemes, so long as the coupling between the internal and relative external DoF of the protein and ligand is weak when the ligand is decoupled. However, increasingly negative binding free energies would be expected as more restrictive restraints are used and with increasing differences between the *apo* and *holo* conformations of the binding site, and the magnitude of the error may be be difficult to predict.

## 5 Conclusion

The free energies of binding for MIF-180 to MIF calculated with varied sets of Boresch restraints were fairly self-consistent and in good agreement with experiment. However, removal of the orientational restraints produced estimates which were up to approximately 4 kcal mol^–1^ more negative, likely because the ligand failed to sample all relevant orientations as the LJ interactions were removed. It was found that the calculations were highly sensitive to the sampling of water in the binding site at intermediate stages of vanishing and that underhydration of the binding site during these stages over as few as 3 λ windows could shift the binding free energy by over 3 kcal mol^–1^ toward more favorable binding.

**Figure 13:**
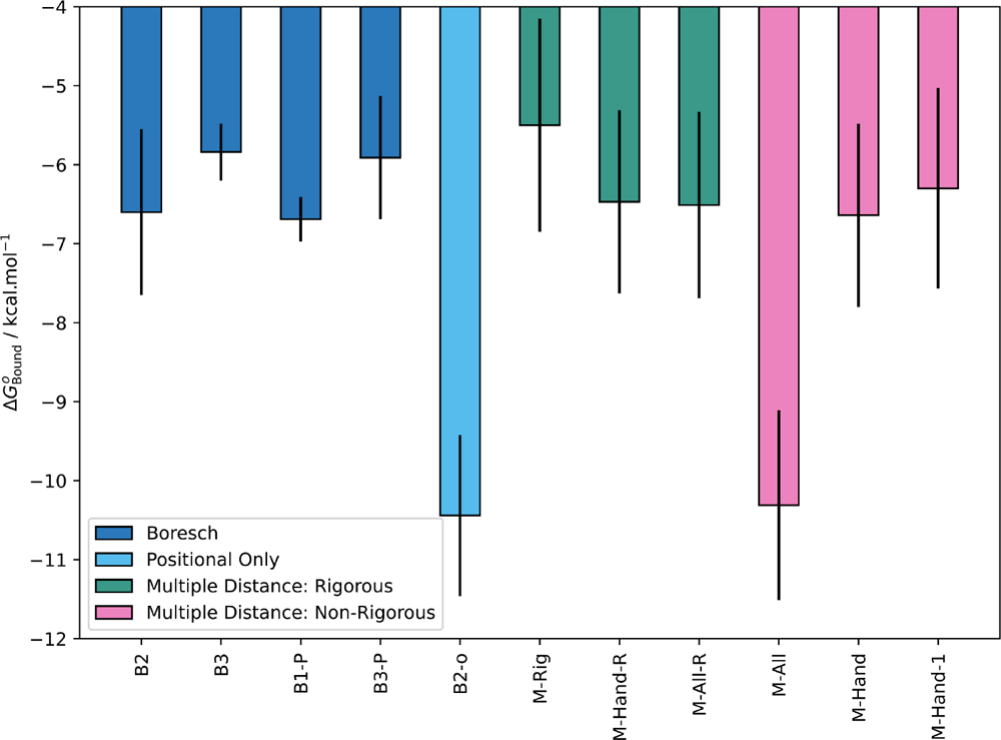
Summary of results for *ΔG*_Bound_^*o*^ obtained for binding pose A using a variety of restraints schemes. Uncertainties are 95% confidence intervals based on the variance of five replicate runs, assuming Gaussian distributions. Results for B1 have been omitted as they appeared to be more susceptible to water sampling issues. For B3-P, run 1 was excluded from the average due to undersampling of water in binding site during the vanishing stage.

Instabilities inherent to the Boresch restraint scheme were highlighted by the failure of several simulations, even with sensible restraint parameters which imposed a minimum energy penalty of around 10 *k*_B_*T* for collinear anchor points. The use of multiple distance restraints offers an alternative restraint scheme which lacks the instabilities of Boresch restraints and may improve convergence during decoupling by allowing greater restriction of ligand movements. The theory of multiple distance restraints was discussed, and a rigorous implementation of the multiple distance restraints scheme was proposed. This utilized intramolecular rigidification of anchor points to prevent coupling between the internal and external DoF. This was shown to produce free energy estimates in good agreement with the Boresch restraints, at least within the large uncertainties encountered with this system (Figure 13). This scheme incurred a substantial additional computational cost over Boresch restraints because aggressive rigidification was required to counter the strong intermolecular restraints, but the scheme may offer benefits where more permissive intermolecular restraints are used.

Another rigorous implementation of the multiple distance restraints scheme was tested, which involved releasing the multiple restraints to a single distance restraint after decoupling. In contrast to calculations performed entirely without orientational restraints, this scheme produced free energy estimates in good agreement with the Boresch restraints scheme, at a reduced computational cost compared to the scheme employing intramolecular restraints. The additional computational cost compared to Boresch restraints is expected to be less than a third of the vanish stage unless very many strong intermolecular restraints are used. Additional costs associated with rigorous multiple distance restraints schemes may be compensated for by convergence benefits in some systems, although that was not demonstrated in this work. The mean *ΔG*_Bound_^*o*^ calculated based on pose A with rigorous implementations of multiple distance restraints (−6.16 ± 1.43 kcal mol^–1^) was in close agreement with the mean result calculated with Boresch restraints (−6.28 ± 0.49 kcal mol^–1^).

Finally, a nonrigorous implementation of the multiple distance restraints scheme was tested, which relied on the assumption of negligible coupling between the internal and external DoF. With strong restraints, this assumption was violated and excessively negative free energies of binding were calculated, but quantities close to the rigorous estimates were obtained with sufficiently permissive restraints. However, it may be difficult to predict the magnitude of the error introduced by this implementation.

The dominant source of uncertainty in these calculations appeared to be the sampling of water, and no convergence benefits were demonstrated with multiple distance restraints over Boresch restraints. Future work may investigate whether convergence benefits are observed in systems where ligand conformational sampling is the dominant source of uncertainty. Further comparison against a wider range of restraint schemes over a variety of systems is also the subject of future work.

In summary, this work demonstrates that absolute binding free energies equivalent to those obtained with Boresch restraints can be calculated using multiple distance restraints. This framework is in principle more stable and may offer convergence benefits during decoupling, although this must be balanced against the additional computational cost incurred by the extra stages required for the rigorous schemes. However, a multiple distance restraints scheme utilizing many flat-bottomed potentials to closely restrain the ligand with minimal disturbance of the interacting system may allow the restraining stage to be neglected, as with the DBC restraint,^29^ while improving the convergence of the decoupling stages when the ligand is flexible. This work discussed the theory and demonstrated proof-of-principle of rigorous multiple distance restraint schemes; future work may investigate whether the scheme can offer performance benefits.

## Data Availability

Example input files, restraint selection code, and modified version of Sire required for the rigorous multiple distance restraints calculations are available on GitHub at michellab/Multiple-Distance-Restraints..

## Supporting Information Available

Discussion of the theoretical requirement for receptor–ligand restraints, derivation of eq 4, diagram of the *syn* and *anti* conformations of MIF-180, parameters for all restraint sets, RMSFs of residues containing anchor points for Boresch restraints, discussion of challenges and potential improvements related to the restraint selection procedure, discussion of the force constant fitting procedure, convergence of free energies with simulation time, bound vanish stage PMF and plot of number of waters in the binding site for B3-P, plot of the number of waters in the binding site during the bound vaish stage for B2-o, comparison of the results without orientational restraints to the literature, results showing convergence of restraint correction for multiple distance restraints schemes, overlap matrices for selected multiple distance restraint protocols, and convergence of the free energy of preorganization for M-Rig. (PDF)
