# Supplementary material for: Comparison of Receptor–Ligand Restraint Schemes for Alchemical Absolute Binding Free Energy Calculations
Source: J Chem Theory Comput. 2023 Jun 7;19(12):3686–704. doi: 10.1021/acs.jctc.3c00139 (PMC10308817; doi:10.1021/acs.jctc.3c00139)
Supplement: Supplementary file 1 — ct3c00139_si_001.pdf [file ct3c00139_si_001.pdf]

Comparison of Receptor-Ligand Restraint Schemes for Alchemical Absolute Binding Free Energy Calculations: Supplementary Information

Finlay Clark,[†] Graeme Robb,[‡] Daniel J. Cole,[¶] and Julien Michel[*,†]

†*EaStCHEM School of Chemistry, University of Edinburgh, David Brewster Road, Edinburgh EH9 3FJ, United Kingdom*

‡*Oncology R&D, AstraZeneca, Cambridge CB4 0WG, United Kingdom*

¶*School of Natural and Environmental Sciences, Newcastle University, Newcastle upon Tyne NE1 7RU, United Kingdom*

E-mail: julien.michel@ed.ac.uk

S1 Restraints are not Necessary to Prevent Errors Arising from an Incorrect Definition of the Bound State for Strong Binders

When no receptor-ligand restraints are used in an ABFE calculation, the implicit definition of the bound state includes configurations where the ligand is anywhere in the entire simulation box relative to the receptor. This section shows that even in the limit of perfect sampling, this introduces errors in computed binding free energies for weak binders, but not for sufficiently strong binders in a sufficiently small simulation box.

In a hypothetical alchemical calculation with perfect sampling, receptor-ligand restraints

would only be required for weak binders. This is because for strong binders, the free energy of the macrostate where the interacting ligand is confined to the binding site is almost identical to the free energy of the macrostate where the ligand is allowed to explore the entire simulation box. This argument is equivalent to that of Gilson et al.,[1] who showed that the standard chemical potential is insensitive to the cutoff function (e.g. restraints) for strong binders. Intuitively, the probability of finding the ligand outside the binding site when it is fully interacting is negligible, and therefore including the unbound configurations makes only a negligible difference to the definition of the state when the box is not extremely large. In such a hypothetical simulation with a strong binder, perfect sampling, and no restraints, the ligand would be decoupled while it was allowed to explore the entire simulation box in the presence of the receptor. The standard state dependence would then be included by correcting the binding free energy by $-k_{\mathrm{B}}T\ln\frac{V_{\mathrm{Box}}}{V^o}$, where V_{Box} is the volume of the simulation box, and $V^o = 1660$ Å^3 is the standard state volume. The free energy obtained would be the same as when restricting ligand sampling to the binding site (assuming that this definition included all low energy configurations,[1] or that restraints were introduced starting with the ligand kinetically trapped in the binding site and the free energy cost of turning them on was accounted for), as done in this work.

In contrast, for a weak binder, the free energy of the macrostate where the interacting ligand can explore the entire simulation box is substantially different to the macrostate where the ligand only samples the binding site. Therefore, starting from the macrostate where the ligand may sample the entire simulation box introduces substantial error, and restraints are required to ensure that the ligand only samples the binding site. In this case, restraints are required for the correct binding site definition.

To illustrate this, the free energies obtained from these hypothetical simulations can be calculated A) restricting the ligand sampling to the binding site ($\Delta G^o_{\mathrm{Bind,\ Site}}$) and B) allowing the ligand to sample the entire simulation box ($\Delta G^o_{\mathrm{Bind,\ Box}}$). $\Delta G^o_{\mathrm{Bind,\ Site}}$ gives the difference in free energy between the fully-interacting ligand in the binding site and the

fully interacting ligand in a water box of volume 1660 Å^3. Therefore, assuming negligible interaction of the ligand and receptor outside of the binding site, and negligible volume of the receptor compared to the box volume, the two free energies of binding are related by

$$\Delta G^o_{\text{Bind, Box}} = -k_\text{B}T\ln\left(\exp^{-\frac{\Delta G^o_{\text{Bind, Site}}}{k_\text{B}T}} + \frac{V_\text{Box}}{V^o}\right).$$

Taking $V_\text{Box} = 512000$ Å^3 (side length of 80 Å) and $T = 298$ K, the two binding free energies can be compared (Figure S1):

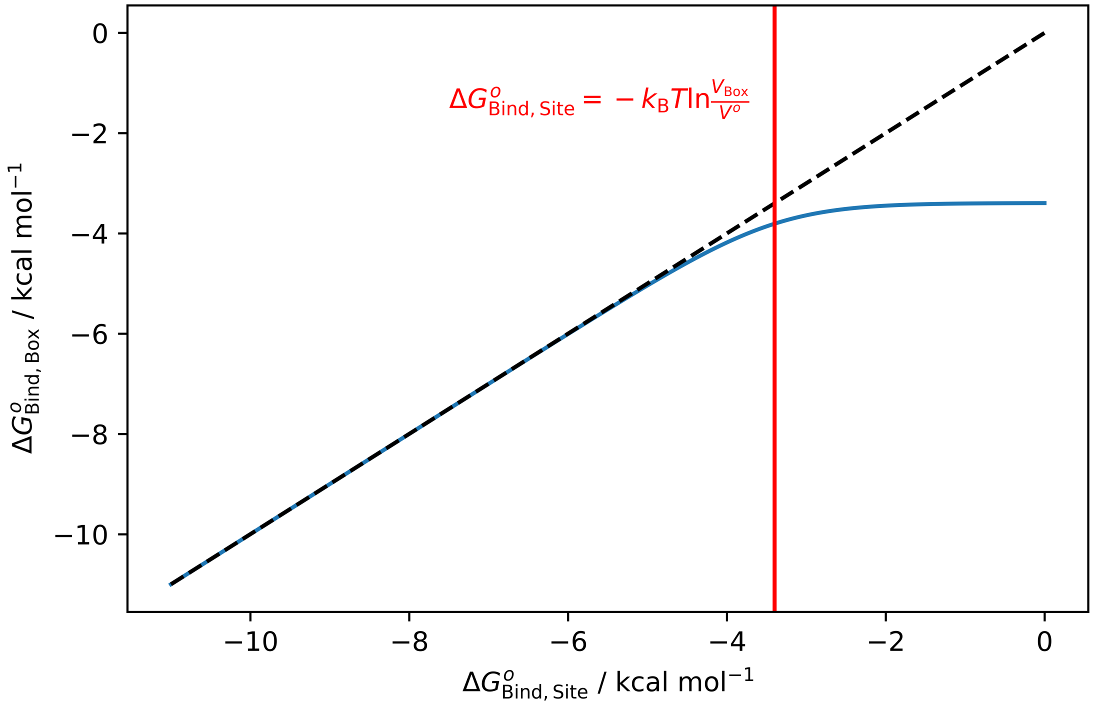

Figure S1: $\Delta G^o_{\text{Bind, Site}}$ against $\Delta G^o_{\text{Bind, Box}}$ for a box size of 51200 Å^3 at 298 K. The dashed line shows $\Delta G^o_{\text{Bind, Site}} = \Delta G^o_{\text{Bind, Box}}$. For $\Delta G^o_{\text{Bind, Site}} < -k_\text{B}T\ln\frac{V_\text{Box}}{V^o}$, there is a negligible difference between the two free energies, and thus restraints are not required to define the binding site.

When $\Delta G^o_{\text{Bind, Site}} > -k_\text{B}T\ln\frac{V_\text{Box}}{V^o}$, $\Delta G^o_{\text{Bind, Box}}$ tends to $-k_\text{B}T\ln\frac{V_\text{Box}}{V^o} = -3.4$ kcal mol^{-1}, the entropy of releasing a water from a box of volume 1660 to 51200 Å^3. For $\Delta G^o_{\text{Bind, Site}} < -k_\text{B}T\ln\frac{V_\text{Box}}{V^o}$, the difference between the two free energies, $\Delta G^o_{\text{Bind, Site}}$ and $\Delta G^o_{\text{Bind, Box}}$, is negligible and thus restraints are not required to define the binding site. Thus, in the case

of strong binders, receptor-ligand restraints are only required to prevent sampling issues (for example the ligand would have to sample outside the binding site as soon as the unbound state began to contribute significantly to the configurational integral).

S2 Derivation of a General Expression for The Standard Free Energy of Releasing Receptor-Ligand Restraints

The free energy of releasing the restraint on the decoupled ligand is given by the ratio of configurational integrals of states 3 and 4 as defined in the main text (Figure 2)

$$\Delta G_{\text{Release}} = -k_{\text{B}}T \ln \frac{Z_{\text{State 3}}}{Z_{\text{State 4}}}, \tag{S1}$$

where k_{B} is the Boltzmann constant, T is the temperature, and $Z_{\text{State 3}}$ and $Z_{\text{State 4}}$ are the configurational integrals for states 3 and 4. It can be shown (Equation 10 of Boresch et al.[2]) that this can be written as

$$\Delta G_{\text{Release}} = -k_{\text{B}}T \ln \frac{Z_{\text{R, Free}} Z_{\text{L, Free}}}{Z_{\text{C}}}, \tag{S2}$$

where "R, Free", "L, Free", and C denote the configurational integrals for the free receptor, the free decoupled ligand, and the complex where there are restraints between the decoupled ligand and the receptor, respectively. Note that $Z_{\text{R, Free}}$ and Z_{C} include contributions from the interaction of the receptor with the solvent and involve integration over the solvent degrees of freedom (DoF), while $Z_{\text{L, Free}}$ does not include solvent interactions or integration over the solvent degrees of freedom. By integrating out the six external degrees of freedom, equation S2 can be rewritten as

$$\Delta G_{\text{Release}} = -k_{\text{B}}T \ln \frac{\tilde{Z}_{\text{R, Free}} \tilde{Z}_{\text{L, Free}} V_{\text{Box}} 8\pi^2}{\tilde{Z}_{\text{C}}}, \tag{S3}$$

where V_{Box} is the volume of the simulation box and $\tilde{Z}$ denotes integration over the $3N-6$ internal degrees of freedom, where N is the number of atoms in the ligand for $\tilde{Z}_{\text{L, Free}}$, the number of atoms in the receptor and water box for $\tilde{Z}_{\text{R, Free}}$, or the number of atoms in the

restrained receptor-decoupled ligand complex and water box for $\tilde{Z}_{\mathrm{C}}$. Equation S3 stresses that $\Delta G_{\mathrm{Release}}$ is dependent on the box size, which is undesirable. As discussed by Gilson et al.,[1] a correction must be applied in order to yield the standard absolute binding free energies. This standard state dependence was missing from early "double annihilation" calculations,[3] but is correctly accounted for in the modern "double decoupling" alchemical approach. This correction is applied by replacing V_{Box} with $V^o = 1660\text{Å}^3$, the standard state volume, to yield the standard free energy of releasing the restraints

$$\Delta G^o_{\mathrm{Release}} = -k_{\mathrm{B}}T \ln \frac{\tilde{Z}_{\mathrm{R,\ Free}}\tilde{Z}_{\mathrm{L,\ Free}}V^o 8\pi^2}{\tilde{Z}_{\mathrm{C}}}, \tag{S4}$$

which is independent of the box size. $\tilde{Z}_{\mathrm{C}}$ can be expanded, giving

$$\begin{aligned}\Delta G^o_{\mathrm{Release}} =& -k_{\mathrm{B}}T \ln \tilde{Z}_{\mathrm{R\ Free}}\tilde{Z}_{\mathrm{L\ Free}}V^o 8\pi^2 \\ &+ k_{\mathrm{B}}T \ln \int e^{-\frac{W_{\mathrm{r}}(\mathbf{x}_{\mathrm{Ext}})+W_{\mathrm{R,\ Comp.}}(\mathbf{x}_{\mathrm{Ext}})+W_{\mathrm{L,\ Comp.}}(\mathbf{x}_{\mathrm{Ext}})}{k_{\mathrm{B}}T}} |\mathbf{J}| \mathrm{d}\mathbf{x}_{\mathrm{Ext}}\end{aligned} \tag{S5}$$

where $W_{\mathrm{r}}(\mathbf{x}_{\mathrm{Ext}})$ and $W_{\mathrm{L,\ Comp.}}(\mathbf{x}_{\mathrm{Ext}})$ are the potentials of mean force (PMFs) associated with the receptor-ligand restraint energy and the decoupled ligand internal energy (as part of the complex), respectively. $W_{\mathrm{R,\ Comp.}}(\mathbf{x}_{\mathrm{Ext}})$ is the PMF of the receptor internal energy (as part of the complex) and the remaining contributions to the total system energy. These are with respect to the six relative external degrees of freedom of the receptor and ligand, which are contained in the vector $\mathbf{x}_{\mathrm{Ext}}$. These PMFs result from integration over the $3N-12$ remaining internal degrees of freedom of the complex. The form of the Jacobian determinant, $|\mathbf{J}|$, depends on the coordinate transformation used to extract the relative external degrees of freedom from the internal degrees of freedom of the system.

$W_{R,\mathrm{Comp.}}(\mathbf{x}_\mathrm{Ext})$ and $W_{L,\mathrm{Comp.}}(\mathbf{x}_\mathrm{Ext})$ can be expanded to yield

$$\begin{aligned}\Delta G^o_\mathrm{Release} =& - k_\mathrm{B}T \ln \tilde{Z}_\mathrm{R\ Free}\tilde{Z}_\mathrm{L\ Free}V^o 8\pi^2 \\ &+ k_\mathrm{B}T \ln \int e^{-\frac{W_\mathrm{r}(\mathbf{x}_\mathrm{Ext})}{k_\mathrm{B}T}} \times \\ &\quad e^{-\frac{\Delta_1 W_\mathrm{R,\ Comp.}(\mathbf{x}_\mathrm{Ext,0}) + \Delta_2 W_\mathrm{R,\ Comp.}(\mathbf{x}_\mathrm{Ext}) + \tilde{G}_\mathrm{R,\ Free}}{k_\mathrm{B}T}} \times \\ &\quad\quad e^{-\frac{\Delta_1 W_\mathrm{L,\ Comp.}(\mathbf{x}_\mathrm{Ext,0}) + \Delta_2 W_\mathrm{L,\ Comp.}(\mathbf{x}_\mathrm{Ext}) + \tilde{G}_\mathrm{L,\ Free}}{k_\mathrm{B}T}} |\mathbf{J}| \mathrm{d}\mathbf{x}_\mathrm{Ext}, \end{aligned} \tag{S6}$$

where the vector $\mathbf{x}_\mathrm{Ext,0}$ contains the values of the relative external degrees of freedom at which the PMF $W_\mathrm{r}(\mathbf{x}_\mathrm{Ext})$ has its minimum. $\tilde{G}_\mathrm{L,\ Free}$ is the free energy of the internal degrees of freedom of the decoupled ligand in the absence of receptor-ligand restraints, $\Delta_1 W_{L,\mathrm{Comp.}}(\mathbf{x}_\mathrm{Ext,0})$ is the free energy of "preorganising" the ligand intramolecular degrees of freedom when the restraint energy is at its minimum, and $\Delta_2 W_\mathrm{L,\ Comp.}(\mathbf{x}_\mathrm{Ext}) = W_\mathrm{L,\ Comp.}(\mathbf{x}_\mathrm{Ext}) - \Delta_1 W_{L,\mathrm{Comp.}}(\mathbf{x}_\mathrm{Ext,0}) - \tilde{G}_\mathrm{L,\ Free}$ is the additional free energy penalty from further distortion of the ligand internal degrees of freedom. It is 0 when $\mathbf{x}_\mathrm{Ext} = \mathbf{x}_\mathrm{Ext,0}$. These terms give rise to $\tilde{Z}_\mathrm{L,\ Free}$, $\Delta G_\mathrm{L,\ Preorg.}$, and $\Delta G_\mathrm{L,\ Distort.}$, respectively. There are equivalent terms for the receptor, which also include contributions from solvent interactions. Rewriting Equation S6 gives

$$\begin{aligned}\Delta G^o_\mathrm{Release} =& - k_\mathrm{B}T \ln \tilde{Z}_\mathrm{R\ Free}\tilde{Z}_\mathrm{L\ Free}V^o 8\pi^2 \\ &+ k_\mathrm{B}T \ln \int e^{-\frac{W_\mathrm{r}(\mathbf{x}_\mathrm{Ext})}{k_\mathrm{B}T}} |\mathbf{J}| \mathrm{d}\mathbf{x}_\mathrm{Ext} + k_\mathrm{B}T \ln \tilde{Z}_\mathrm{R,\ Free}\tilde{Z}_\mathrm{L,\ Free} \\ &- \Delta G_\mathrm{R,\ Preorg.} - \Delta G_\mathrm{L,\ Preorg.} - \Delta G_\mathrm{R,\ Distort.} - \Delta G_\mathrm{L,\ Distort.} \\ =& - k_\mathrm{B}T \ln V^o 8\pi^2 + k_\mathrm{B}T \ln \int e^{-\frac{W_\mathrm{r}(\mathbf{x}_\mathrm{Ext})}{k_\mathrm{B}T}} |\mathbf{J}| \mathrm{d}\mathbf{x}_\mathrm{Ext} - \Delta G_\mathrm{Preorg.} - \Delta G_\mathrm{Distort.}, \end{aligned} \tag{S7}$$

where $\Delta G_\mathrm{Preorg.}$ and $\Delta G_\mathrm{Distort.}$ are the sums of the preorganisation and distortion terms for the receptor and the ligand.

S3 The *syn* and *anti* conformations of MIF180

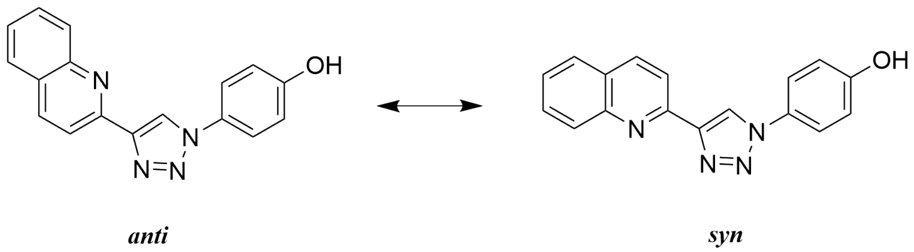

Figure S2: The *syn* and *anti* conformations of MIF180.

S4 Parameters for Boresch Restraints

Table S1: Parameters for Boresch restraints, as labelled in Figure 3. K refers to a force constant and 0 denotes and equilibrium value.

	**Restraint Set**					
**Parameter**	**B1**	**B2**	**B3**	**B1-poseB**	**B1-P**	**B3-P**
c (index)	4949	937	51	4946	965	605
b (index)	4944	933	47	4942	961	601
a (index)	4946	935	49	4944	963	603
A (index)	11	10	12	14	5	6
B (index)	2	13	6	4	3	9
C (index)	3	20	19	5	14	12
r_0 (Å)	5.92	8.14	7.84	8.69	6.94	8.48
$\theta_{\mathrm{A},0}$ (rad)	1.85	2.06	0.81	1.54	0.66	1.24
$\theta_{\mathrm{B},0}$ (rad)	1.59	1.89	1.74	1.52	2.10	2.09
$\phi_{\mathrm{A},0}$ (rad)	-0.30	1.68	2.59	-1.22	-0.14	2.55
$\phi_{\mathrm{B},0}$ (rad)	-1.55	1.52	-1.20	2.80	-1.32	-0.20
$\phi_{\mathrm{C},0}$ (rad)	2.90	0.20	2.63	-0.29	-0.11	2.15
K_r (kcal mol^{-1} Å^{-2})	25.49	10.92	10.25	12.25	14.27	6.32
K_{θ_A} (kcal mol^{-1} rad^{-2})	66.74	126.83	49.44	90.22	91.57	73.31
K_{θ_B} (kcal mol^{-1} rad^{-2})	38.39	98.43	99.26	115.70	100.27	63.90
K_{ϕ_A} (kcal mol^{-1} rad^{-2})	215.36	189.35	51.25	189.91	71.33	68.44
K_{ϕ_B} (kcal mol^{-1} rad^{-2})	49.23	58.81	25.98	42.35	72.02	69.07
K_{ϕ_C} (kcal mol^{-1} rad^{-2})	49.79	100.72	95.41	174.58	71.25	44.54

S5 RMSF of Residues Containing Anchor Points

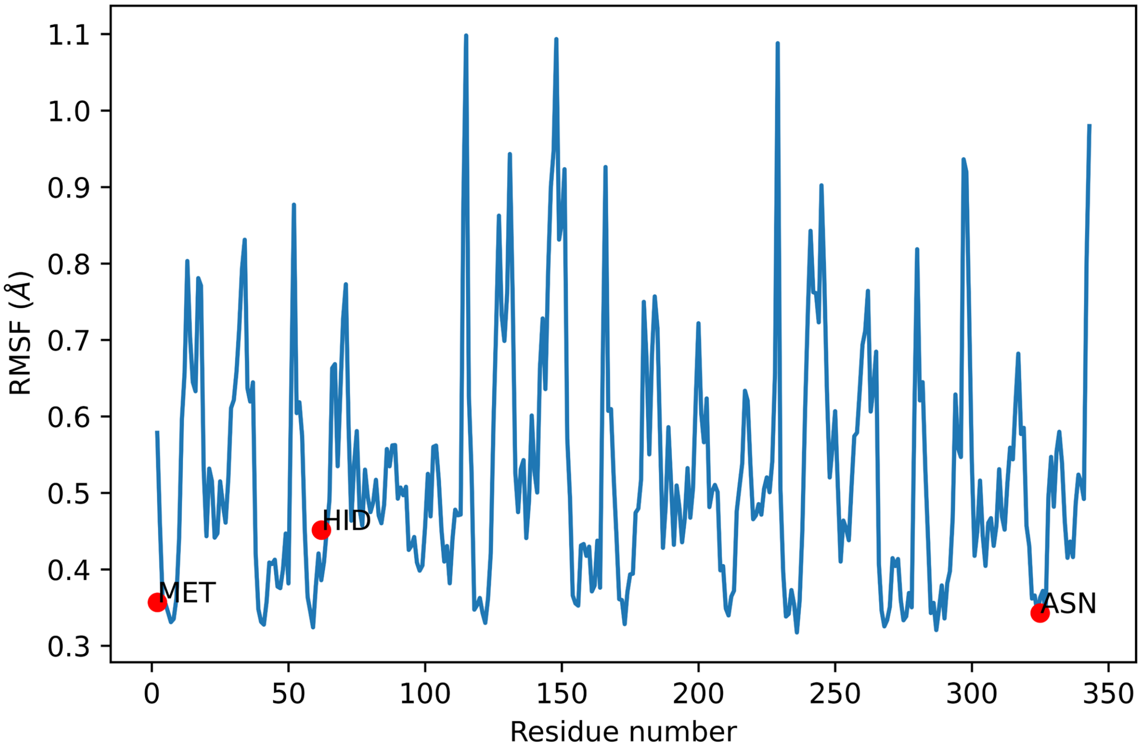

Figure S3: The restraint selection algorithm produces protein anchor points on stable residues. The RMSF of the Cα atom of each residue was calculated over the unrestrained simulation used for restraint selection, after all frames were aligned to the average structure. MET, HID, and ASN residues highlighted in red contain the anchor points used for the B3, B2, and B1 restraints, respectively.

S6 Challenges in Restraint Selection

The selection of Boresch restraints highlighted challenges related to the presence of multiple binding poses and the treatment of symmetry. Fitting the force constants to simulation provides a crude estimate of the PMFs with respect to the Boresch degrees of freedom. Thus, the force constants for the Boresch DoF are effectively doubled during the restraining stage. Under the approximations of Gaussian distributions and no correlation between the Boresch DoF, the free energy of turning on the restraints can be estimated from Equation 7; this should be approximately $-k_BT\ln((2^6)^{1/2}) = -1.23$ kcal mol^{-1} at 298 K in all cases.

This observation allows the identification of issues during the restraining stages. $\Delta G_{\text{Bound, Restrain}} > 5$ kcal mol^{-1} was calculated from initial simulations, which indicated an issue with binding poses which slowly interconverted on the timescale of simulation (Figure 6). This resulted from an almost immediate change to an alternate binding pose from the input structure during the simulation used to fit the restraints. Because this pose persisted throughout the unrestrained simulation, relatively tight force constants were selected, resulting in the calculation of large free energies of restraining to the alternative pose starting from the original input structure.

Subsequent calculations were run by fitting restraints for a single binding pose, and rerunning simulations from where the alternate pose was sampled. Reruns were only required at low values of λ during the restraining calculations as sampling of the alternate pose was prevented by the engaged restraints. These switches were easily identified by checking for large jumps in $\mathrm{d}H/\mathrm{d}\lambda$, as suggested by Baumann et al..[4]

A completely automated restraint selection algorithm would account for symmetry, which the method used here does not. The flip of the phenol group in MIF180 occurs infrequently on the timescale of the simulations, and does not occur at all during the early stages of decoupling in the bound leg. When transitions between symmetrical energy minima (e.g. flip of the phenol) are not sampled, and the symmetry of the minima is not broken by the introduction of restraints, some authors have applied symmetry corrections.[5] However, this

is unnecessary,[6] because free energy changes are obtained from distributions of energies,[7] or their gradients with respect to λ.[8] These distributions are identical regardless of whether one or all of the symmetrical minima are sampled, and therefore the free energy estimates do not change. Thus, no symmetry corrections are required in general here (beyond those accounting for the restraint of the ligand to a single binding site when there are three identical binding sites present per protein).

However, when the receptor-ligand restraints break this symmetry, this must be accounted for. The symmetric wells must either be sampled in equilibrium as the symmetry is broken (the phenol must sample both orientations in equilibrium as the restraints are turned on), or in the case where only one minimum is sampled (the phenol does not flip), a correction must be applied.[6] The latter occurs for the B1 restraints, which impose a large energy penalty for the flip of the phenol. The phenol does not flip during the restraining simulations and hence the associated correction is $-k_BT\ln 2 = -0.41$ kcal mol^{-1}. However, placing restraints on the phenol necessitates equilibrium sampling about the triazole-phenol dihedral during decoupling, which is not the case for the other restraints (because for the other restraints, rotation around this bond results in transitions between symmetrical energy minima). The requirement for both a symmetry correction and equilibrium sampling about the triazole-phenol dihedral could be removed by introducing a flat-bottomed restraint to prevent the phenol flip, as suggested by Wang et al..[9] This would only be required during the bound leg simulations. Alternatively, symmetry-adapted restraints could be used.[10]

It was observed that several of the restraint sets selected had large equilibrium r distances (Table S1) of around 8 Å. It is possible that this resulted from a bias of the restraint selection algorithm towards large values of r as a result of scoring by minimum variance of the Boresch DoF alone: for the same absolute movement of anchor points a or A normal to the vector between anchors A and a, the variation in θ_A and θ_B will be smaller when r is greater. A more natural choice of "score" for prospective restraints may be the total configurational volume accessible in the decoupled state, calculated using Equation 7 for the force constants fitted

to simulation. A smaller accessible configurational volume would score higher. The objective is the same as with scoring by total variance: to select restraints which mimic the strongest receptor-ligand interactions. The two metrics are closely related, because the force constants calculated from the variances of the DoF are used to calculate the accessible configurational volume. However, as a result of the Jacobian terms in Equation 7, this metric is more consistent between restraint sets with different equilibrium distances and angles, avoiding issues with bias as discussed above. It would also be sensible to exclude groups which may be stable while interacting with the ligand, but which may be substantially more mobile when the ligand is decoupled, for example by restricting the protein anchor atoms to the backbone and Cα atoms.[11]

S7 Fitting of Restraint Force Constants

Restraint force constants were fitted by measuring the distributions of the DoF to be restrained in the absence of any restraints, for the fully interacting complex. At equilibrium, a harmonic oscillator produces a Gaussian distribution about its equilibrium value

$$P(x) = \sqrt{\frac{K}{2k_{\mathrm{B}}T\pi}} e^{-\frac{K(x-x_0)^2}{2k_{\mathrm{B}}T}}, \tag{S8}$$

where $P(x)$ is the probability of the restrained DoF taking the value x, x_0 is the mean value of the DoF where no force is applied by the restraint, k_{B} is the Boltzmann constant, T is the temperature in Kelvin, and K is the force constant. Comparing this to the standard expression for a Normal distribution

$$P(x) = \frac{1}{\sqrt{2\pi\sigma^2}} e^{-\frac{(x-\mu)^2}{2\sigma^2}}, \tag{S9}$$

where $\mu = x_0$ and σ^2 is the variance, it can be seen that setting $K = \frac{k_{\mathrm{B}}T}{\sigma^2}$ makes Equations S8 and S9 equivalent.

If the distribution of the DoF about its mean in the fully interacting, non-restrained state is Gaussian, then measuring the variance of this distribution and setting $K = \frac{k_{\mathrm{B}}T}{\sigma^2}$ will mean that when the ligand is decoupled, the distribution of the DoF about its mean will be the same as in the fully interacting, non-restrained state. The force constants were fit in this way to minimise changes to the distributions of the restrained DoF during decoupling, which was intended to enhance convergence. Where only a limited number of DoF can be restrained, as with Boresch restraints, selecting the DoF with the minimum variance in the full-interacting, non-restrained state meant that K was as large as possible, hence the restraints were as restrictive as possible, without having to restrict the movement of an otherwise high-variance DoF, potentially causing convergence issues.

S8 Convergence of Boresch Simulations with Force Constants Fit to Simulation

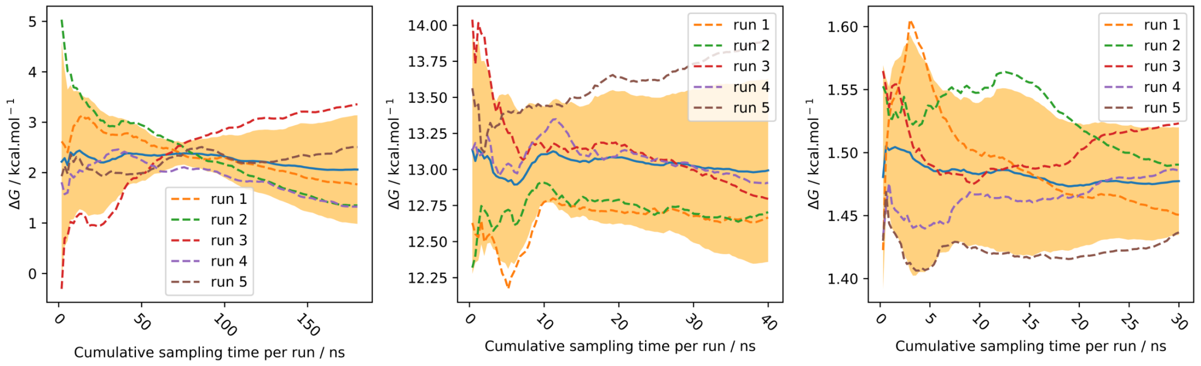

Figure S4: Convergence of the bound leg simulations with B1 with cumulative sampling time per window. From left to right: the vanish, discharge, and restrain stages. Shaded area shows the 95% confidence interval and the solid blue line shows the mean.

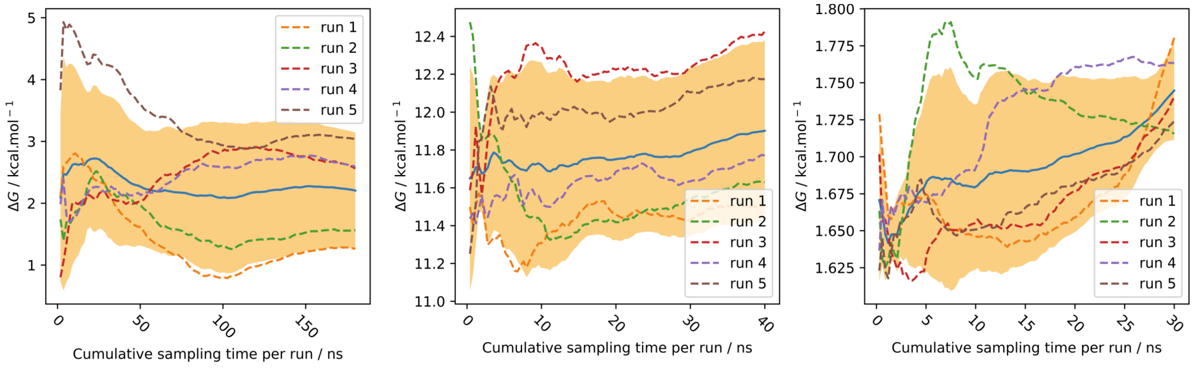

Figure S5: Convergence of the bound leg simulations for B2 with cumulative sampling time per window. From left to right: the vanish, discharge, and restrain stages. Shaded area shows the 95% confidence interval and the solid blue line shows the mean.

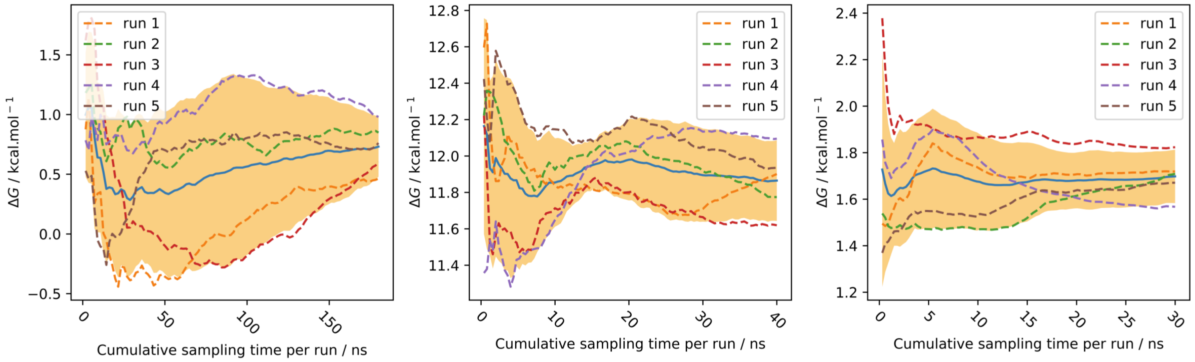

Figure S6: Convergence of the bound leg simulations for B3 with cumulative sampling time per window. From left to right: the vanish, discharge, and restrain stages. Shaded area shows the 95% confidence interval and the solid blue line shows the mean.

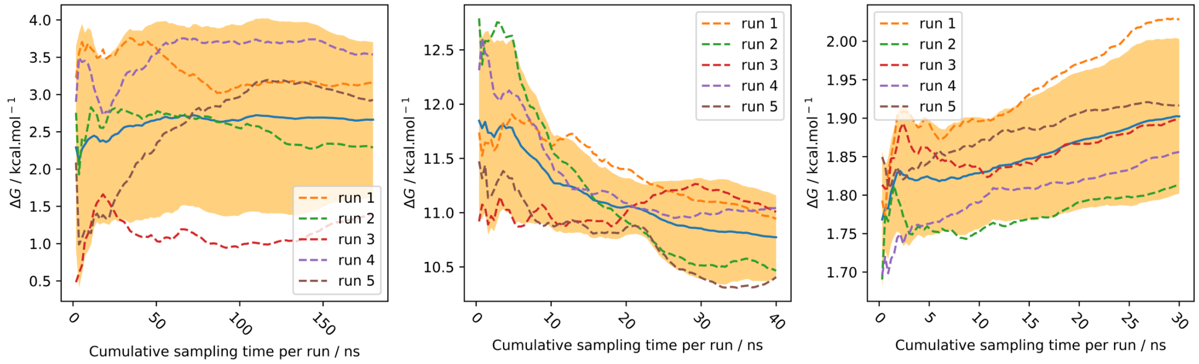

Figure S7: Convergence of the bound leg simulations for B1-poseB with cumulative sampling time per window. From left to right: the vanish, discharge, and restrain stages. Shaded area shows the 95% confidence interval and the solid blue line shows the mean.

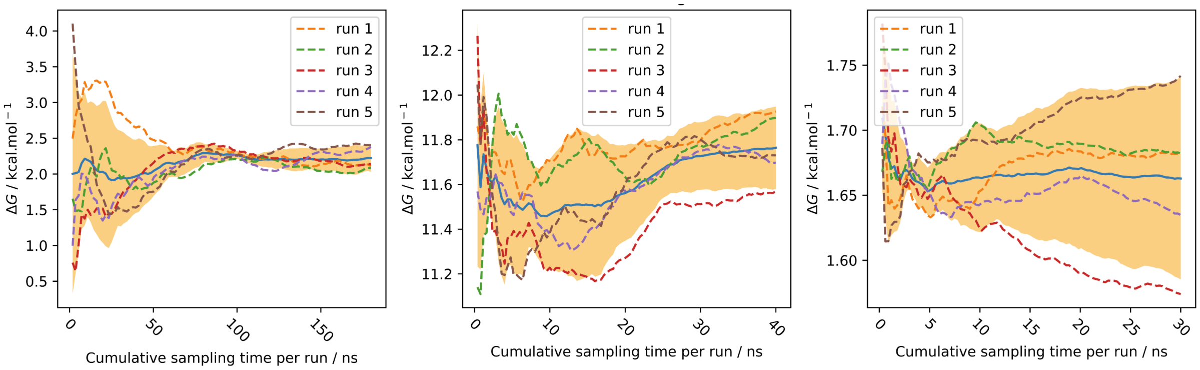

Figure S8: Convergence of the bound leg simulations for B1-P with cumulative sampling time per window. From left to right: the vanish, discharge, and restrain stages. Shaded area shows the 95% confidence interval and the solid blue line shows the mean.

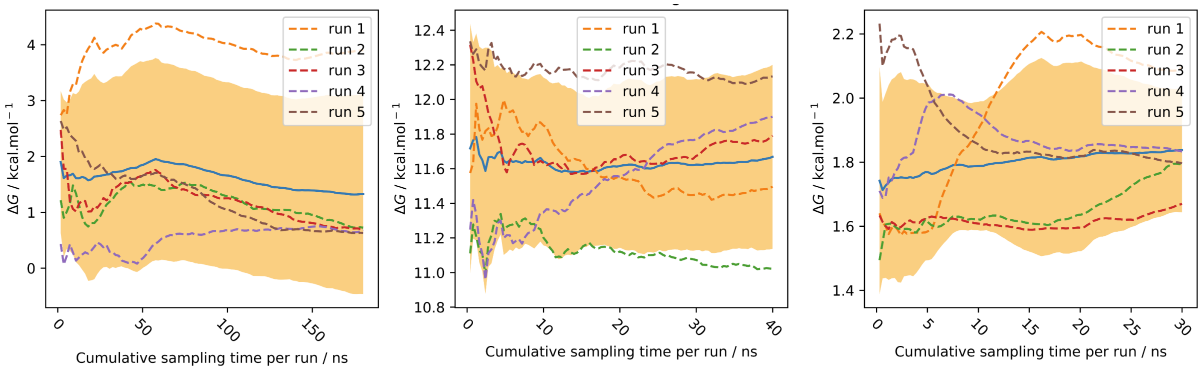

Figure S9: Convergence of the bound leg simulations for B3-P with cumulative sampling time per window. From left to right: the vanish, discharge, and restrain stages. Shaded area shows the 95% confidence interval and the solid blue line shows the mean.

S9 Bound Vanish PMF and Number of Waters in the Binding Site for B3-P

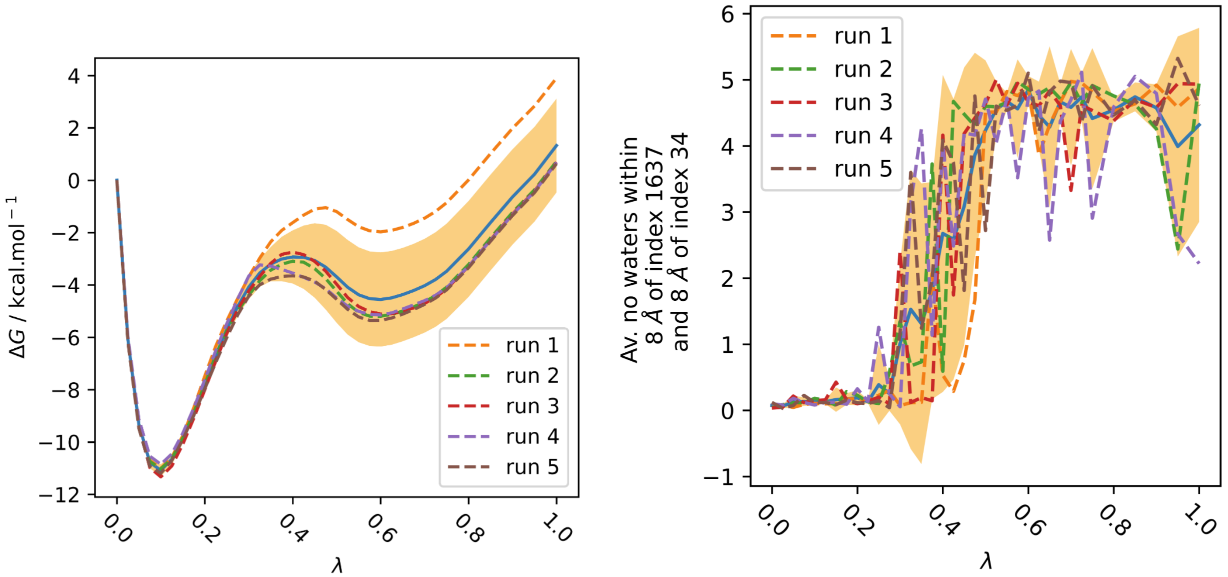

Figure S10: Left - PMF along λ for bound vanish stage for B3-P. Right - Average number of waters in the binding site (as defined as the overlap of two spheres of radius 8 Å centred on the N atom in Pro1A, and CG2 in Val106A - with indices 1637 and 34) against λ during the vanish stage for B3-P. The shaded area shows the 95% confidence interval, and the solid blue line shows the mean. The under-sampling of waters in the binding site over three λ windows near $\lambda = 0.4$ results in the bound vanish PMF for run 1 divering by over 3 kcal mol^{-1}.

S10 Convergence of Free Leg Simulations

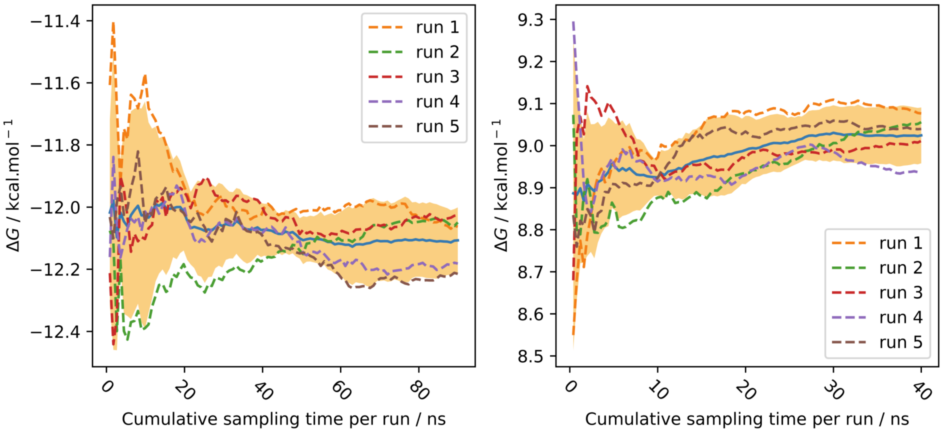

Figure S11: Convergence of the free leg simulations with cumulative sampling time per window. Left: the vanish stage, right: the discharge stage. Shaded area shows the 95% confidence interval.

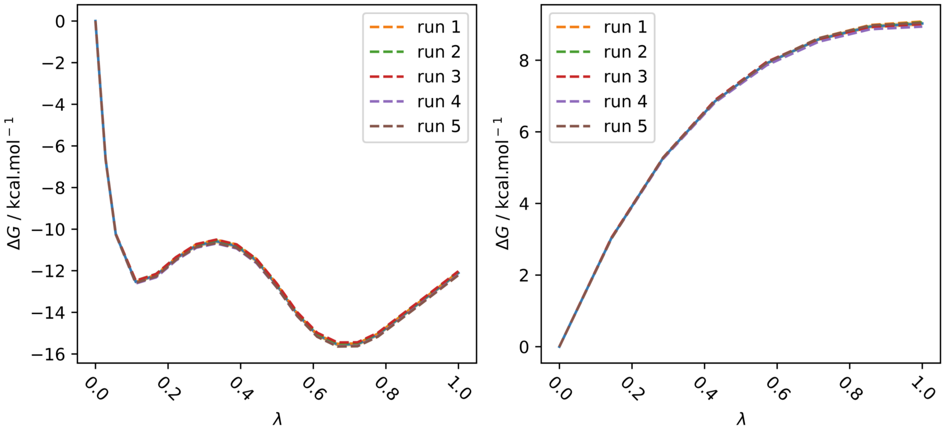

Figure S12: Potentials of mean force along lambda for the free leg simulations.

S11 Convergence of Boresch Simulations with No Orientational Component

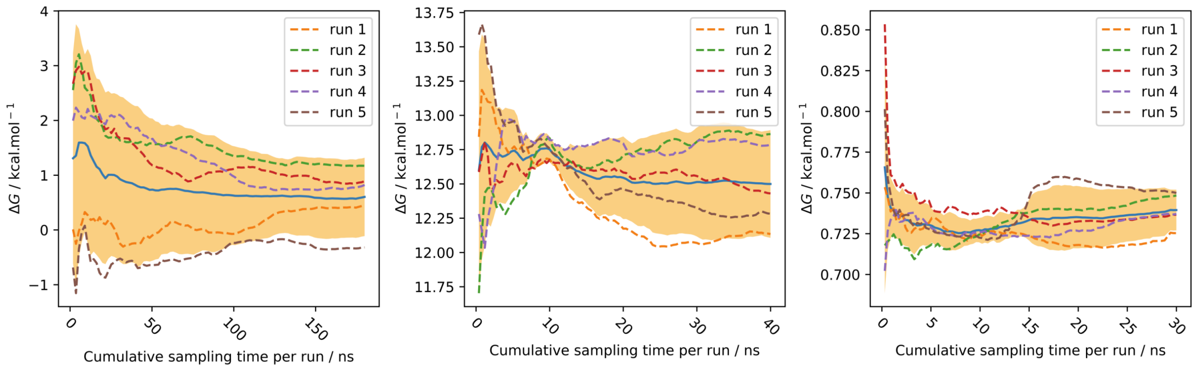

Figure S13: Convergence of the bound leg simulations with B1-o with cumulative sampling time per window. From left to right: the vanish, discharge, and restrain stages. Shaded area shows the 95% confidence interval and the solid blue line shows the mean.

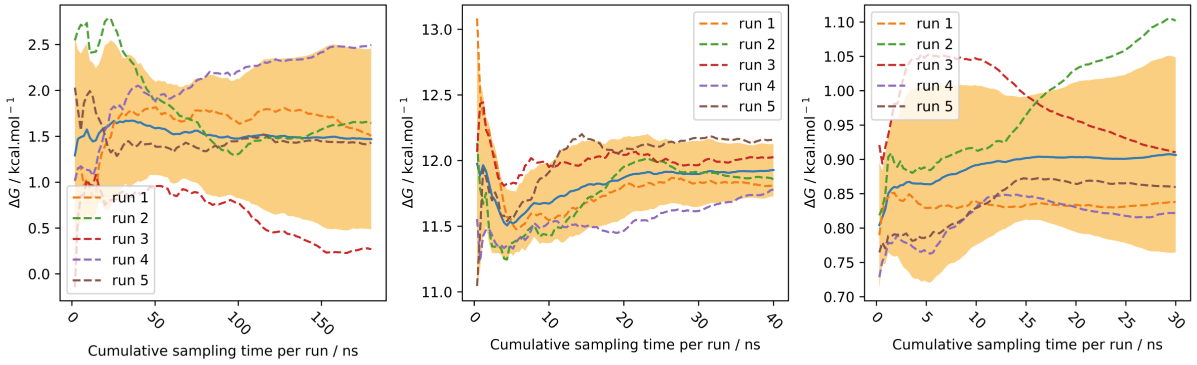

Figure S14: Convergence of the bound leg simulations with B2-o with cumulative sampling time per window. From left to right: the vanish, discharge, and restrain stages. Shaded area shows the 95% confidence interval and the solid blue line shows the mean.

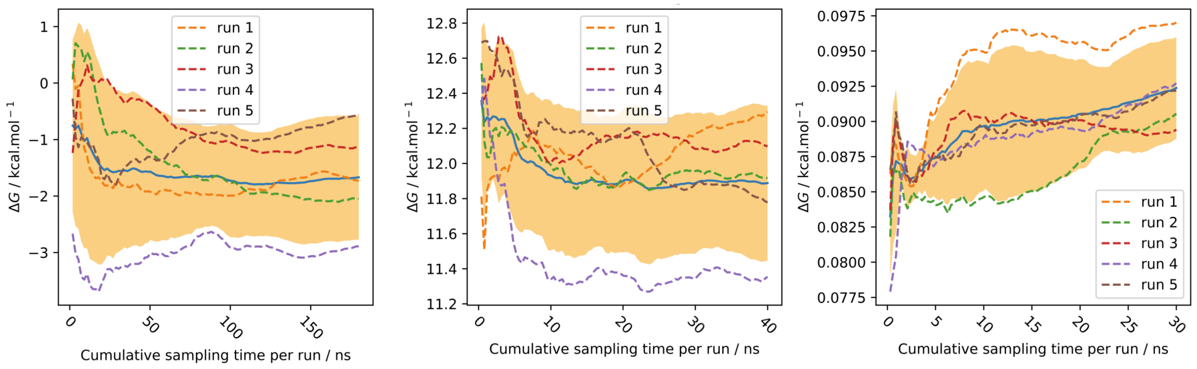

Figure S15: Convergence of the bound leg simulations with B1-d with cumulative sampling time per window. From left to right: the vanish, discharge, and restrain stages. Shaded area shows the 95% confidence interval and the solid blue line shows the mean.

S12 Number of Waters in the Binding Site for B2-o

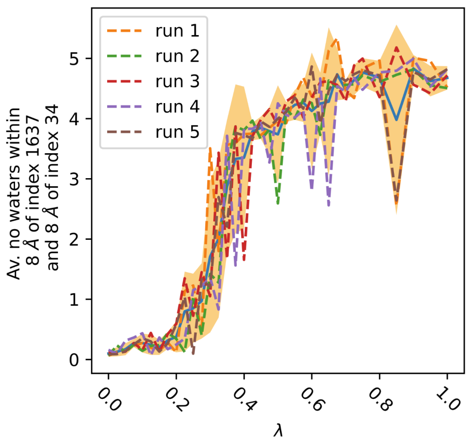

Figure S16: Right - Average number of waters in the binding site (as defined as the overlap of two spheres of radius 8 Å centred on atoms of index 1637 and 34) against λ during the vanish stage for B2-o.

S13 Comparison of Results without Orientational Restraints to the Literature

In this work, it was found that a lack of orientational restraints resulted in an erroneously negative free energy of binding due to failure to sample all relevant orientations during decoupling. Mobley et al. proposed a similar explanation,[5] but found that the offset was towards erroneously positive free energies of binding, which is surprising given the results observed here. However, this may have been due to the incorrect calculation of $-\Delta G^{o}_{\mathrm{Release}}$ in this preceding study.

A value of -5.26 kcal mol^{-1} was calculated for $\Delta G^{o}_{\mathrm{Release}}$ for phenol binding to a T4 lysozyme mutant when using a single distance restraint with a force constant of 10 kcal mol^{-1} Å^{-2} (see $\Delta G^{\mathrm{H_2O}}_{\mathrm{restr}}$ for "No orientational restraints" in Table 1 of Mobley et al.). The the free energy of releasing the restraint is calculated using

$$\Delta G^{o}_{\mathrm{Release}} = k_{\mathrm{B}}T \ln \left(\frac{1}{V^{o}} 4\pi \int_{0}^{r_{\mathrm{Box}}} r^2 e^{-\frac{k(r-r_0)^2}{2RT}} \mathrm{d}r \right)$$

where the terms are as defined for Equation 11. If $r_0 = 0$, a value of -5.27 kcal mol^{-1} is obtained at 298 K, in excellent agreement with the result of Mobley et al.. However, this is incorrect, because $r_0 \neq 0$. r_0 is not stated by Mobley et al., so $r_0 = 3.48$ Å was taken from Table 1 of Boresch et al. for "CL1 FULL", where the same anchors in the protein are used (although the correction does not change dramatically if this distance is changed slightly). This value of r_0 yields a correction of - 1.71 kcal mol^{-1}, giving an overall free energy for the run without orientational restraints of 9.09 ± 0.09 kcal mol^{-1} (subtracting the 0.41 kcal mol^{-1} symmetry correction which is not required when there are no orientational restraints), which is less than 1 kcal mol^{-1} different to the answer obtained with orientation decomposition (10.03 ± 0.05 kcal mol^{-1}). This value would be in even better agreement with a greater r_0. Therefore, it seems likely that in this case there were no large sampling issues caused by

the lack of orientational restraints, and the observed deviation may have resulted from the erroneous use of $r_0 = 0$.

S14 Restraint Dictionaries used for Multiple Distance Restraints Simulations

The format of the restraint dictionaries is {(anchor index 1, anchor index 2): (equilibrium distance, (force constant)/2, flat-bottomed radius), ...}.

S14.1 M-Rig

Intermolecular restraints dictionary:

{(21, 4950): (2.72, 19.55, 0), (11, 4946): (5.92, 12.75, 0), (3, 4909): (3.63, 9.64, 0), (5, 4949): (6.87, 9.28, 0), (4, 971): (4.17, 7.42, 0), (2, 1613): (8.67, 7.07, 0), (14, 963): (5.56, 6.81, 0)}

Intramolecular restraints dictionary for the ligand:

{(21, 11): (1.364, 100, 0), (21, 3): (2.38159, 100, 0), (21, 5): (3.65882, 100, 0), (21, 4): (3.66517, 100, 0), (21, 2): (2.40022, 100, 0), (21, 14): (4.15453, 100, 0), (11, 3): (1.398, 100, 0), (11, 5): (2.42473, 100, 0), (11, 4): (2.41599, 100, 0), (11, 2): (1.398, 100, 0), (11, 14): (2.79524, 100, 0), (3, 5): (1.398, 100, 0), (3, 4): (2.78413, 100, 0), (3, 2): (2.41718, 100, 0), (3, 14): (2.41514, 100, 0), (5, 4): (2.41787, 100, 0), (5, 2): (2.79877, 100, 0), (5, 14): (1.398, 100, 0), (4, 2): (1.398, 100, 0), (4, 14): (1.398, 100, 0), (2, 14): (2.42338, 100, 0)}

Intramolecular restraints dictionary for the protein:

{(4950, 4946): (2.40799, 100, 0), (4950, 4909): (6.23833, 100, 0), (4950, 4949): (1.229, 100, 0), (4950, 971): (7.85489, 100, 0), (4950, 1613): (8.07314, 100, 0), (4950, 963): (9.03817, 100, 0), (4946, 4909): (7.67178, 100, 0), (4946, 4949): (1.522, 100, 0), (4946, 971): (9.71347, 100, 0), (4946, 1613): (8.0947, 100, 0), (4946, 963): (11.01378, 100, 0), (4909, 4949): (6.40042, 100, 0), (4909, 971): (9.2935, 100, 0), (4909, 1613): (11.23339, 100, 0), (4909, 963): (10.82613, 100, 0), (4949, 971): (8.9755, 100, 0), (4949, 1613): (8.49461, 100, 0), (4949, 963): (10.22414, 100, 0), (971, 1613): (7.35912, 100, 0), (971, 963): (2.53581, 100, 0), (1613, 963): (9.33324, 100, 0)}

S14.1.1 M-All

Intermolecular restraints dictionary:

{(21, 4950): (2.72, 19.55, 0), (11, 4946): (5.92, 12.75, 0), (3, 4909): (3.63, 9.64, 0), (5, 4949): (6.87, 9.28, 0), (4, 971): (4.17, 7.42, 0), (2, 1613): (8.67, 7.07, 0), (14, 963): (5.56, 6.81, 0), (20, 959): (4.73, 6.07, 0), (10, 950): (6.68, 5.77, 0), (12, 51): (9.15, 5.64, 0), (18, 4951): (9.52, 5.31, 0), (13, 47): (6.0, 5.02, 0), (19, 49): (8.75, 4.94, 0), (6, 53): (9.8, 3.92, 0), (17, 34): (4.24, 3.64, 0), (16, 45): (7.8, 3.59, 0), (9, 548): (8.14, 2.81, 0), (15, 48): (9.26, 2.74, 0), (7, 584): (8.25, 1.63, 0), (8, 1633): (10.93, 1.6, 0), (0, 1737): (6.64, 1.28, 0), (1, 4914): (10.4, 1.13, 0)}

S14.1.2 M-hand

Intermolecular restraints dictionary was:

{(21, 4950): (2.72, 20, 0.61), (18, 961): (3.09, 20, 0.61), (17, 512): (3.25, 20, 2.06), (19, 512): (3.69, 20, 2.13)}

S15 Convergence of Restraint Correction for Multiple Distance Restraints Schemes

It was confirmed that estimates of $\Delta G^o_{Release}$ had converged with respect to the number of integration points. It was found that evaluating the restraint energy at a relatively large number of orientations was essential to obtain an accurate estimate. Examples are shown below. 50 frames were saved per ns of simulation.

Table S2: Convergence of $\Delta G^o_{\mathrm{Release}}$ for M-Rig with increasing number of grid points used for numerical integration. s is the number of frames to skip between two snapshot evaluations, b is the amount by which the bounding rectangle of the restrained host atoms coordinates is extended in each dimension, d is the edge length of a translational volume element, and o is the number of orientations at which the restraint energy is to evaluated per Euler angle interval ([0,2π] for ϕ and ψ, [0, π] for θ). Insensitivity to b and d was confirmed in preliminary simulations. Uncertainties are the 95 % C.I.s obtained from the variance between 5 replicate runs by assuming Gaussian distributions.

**s**	**b / Å**	**d / Å**	**o**	$\Delta G^o_{\mathbf{Release}}$
1	4	0.25	6	-13.28 ± 4.16
1	4	0.25	12	-10.19 ± 0.28
1	4	0.10	12	-10.19 ± 0.28
1	4	0.25	18	-10.08 ± 0.05
1	4	0.25	24	-10.03 ± 0.07
1	4	0.25	30	-10.02 ± 0.04

Table S3: Convergence of $\Delta G^o_{\text{Release}}$ for M-Hand-1 with increasing number of grid points used for numerical integration. s is the number of frames to skip between two snapshot evaluations, b is the amount by which the bounding rectangle of the restrained host atoms coordinates is extended in each dimension, d is the edge length of a translational volume element, and o is the number of orientations at which the restraint energy is to evaluated per Euler angle interval ([0,2π] for ϕ and ψ, [0, π] for θ). Insensitivity to b and d was confirmed in preliminary simulations. Uncertainties are the 95 % C.I.s obtained from the variance between 5 replicate runs by assuming Gaussian distributions. Note that the final result shown below is slightly different to that shown in the main text, because these checks were performed before a bug affecting flat-bottomed restraints was fixed and all other affected simulations were repeated.

**s**	**b / Å**	**d / Å**	**o**	$\Delta G^o_{\textbf{Release}}$
1	4	0.25	6	-11.41 ± 8.89
1	4	0.25	12	-5.83 ± 0.24
1	4	0.10	12	-5.83 ± 0.24
1	4	0.25	18	-5.74 ± 0.14
1	4	0.25	24	-5.73 ± 0.15
1	4	0.25	30	-5.72 ± 0.15

Table S4: Convergence of $\Delta G^o_{\text{Release}}$ for M-All with increasing number of grid points used for numerical integration. s is the number of frames to skip between two snapshot evaluations, b is the amount by which the bounding rectangle of the restrained host atoms coordinates is extended in each dimension, d is the edge length of a translational volume element, and o is the number of orientations at which the restraint energy is to evaluated per Euler angle interval ([0,2π) for ϕ and ψ, [0, π] for θ). Insensitivity to b and d was confirmed in preliminary simulations. Uncertainties are the 95 % C.I.s obtained from the variance between 5 replicate runs by assuming Gaussian distributions.

**s**	**b / Å**	**d / Å**	**o**	$\Delta G^o_{\textbf{Release}}$
1	4	0.25	6	-61.55 ± 12.12
1	4	0.25	18	-13.68 ± 9.60
1	4	0.25	24	-16.32 ± 0.92
1	4	0.25	30	-15.68 ± 0.37

S16 Convergence of Multiple Distance Restraint Simulations

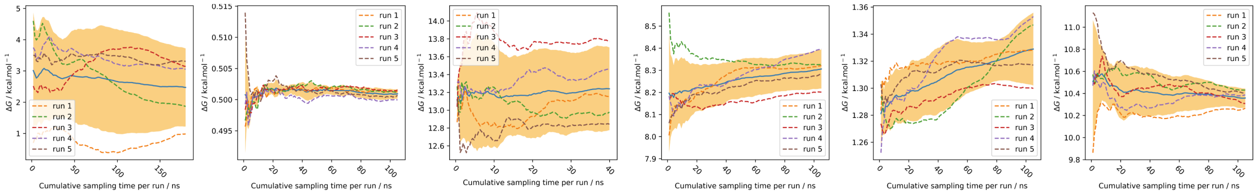

Figure S17: Convergence of the bound leg simulations for M-Rig with cumulative sampling time per window. From left to right: the vanish, rigidify lig, discharge, rigidify complex, restrain, and rigidify recept. stages. Shaded area shows the 95% confidence interval and the solid blue line shows the mean.

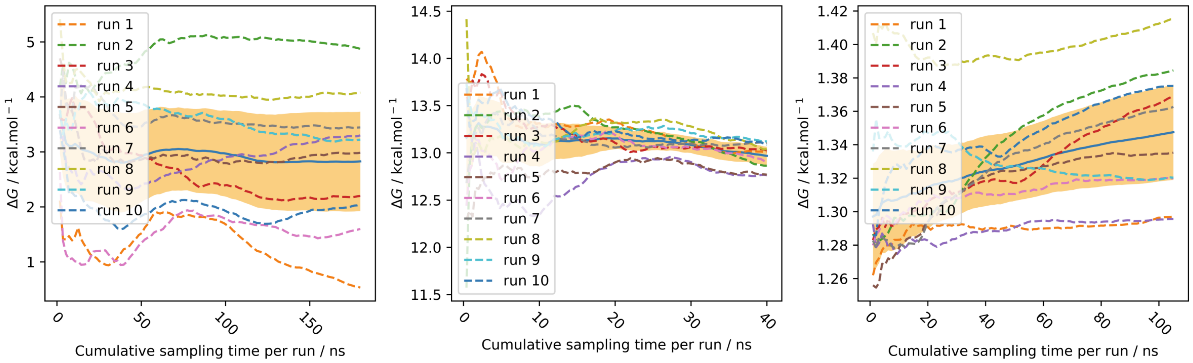

Figure S18: Convergence of the bound leg simulations for M-Rig-N with cumulative sampling time per window. From left to right: the vanish, discharge, and restrain stages. Shaded area shows the 95% confidence interval and the solid blue line shows the mean.

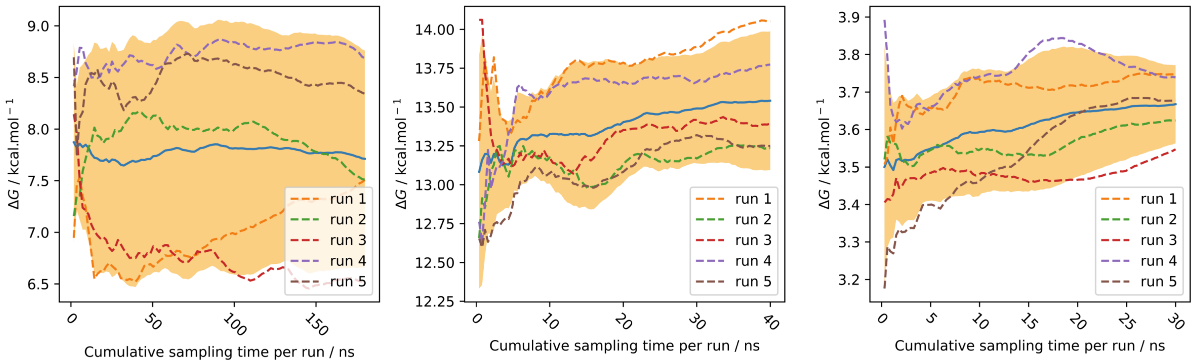

Figure S19: Convergence of the bound leg simulations for M-All with cumulative sampling time per window. From left to right: the vanish, discharge, and restrain stages. Shaded area shows the 95% confidence interval and the solid blue line shows the mean.

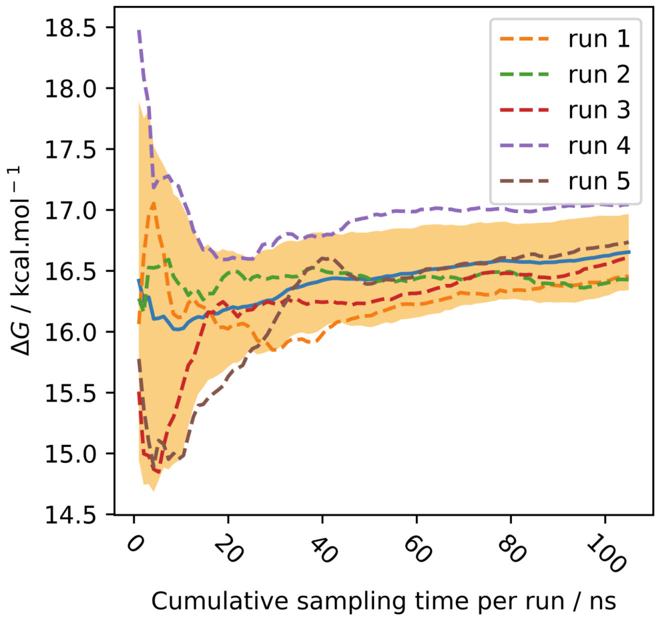

Figure S20: Convergence of the free energy of releasing all distance restraints other than the single strongest instance for M-All-R, with cumulative sampling time per window. Shaded area shows the 95% confidence interval and the solid blue line shows the mean.

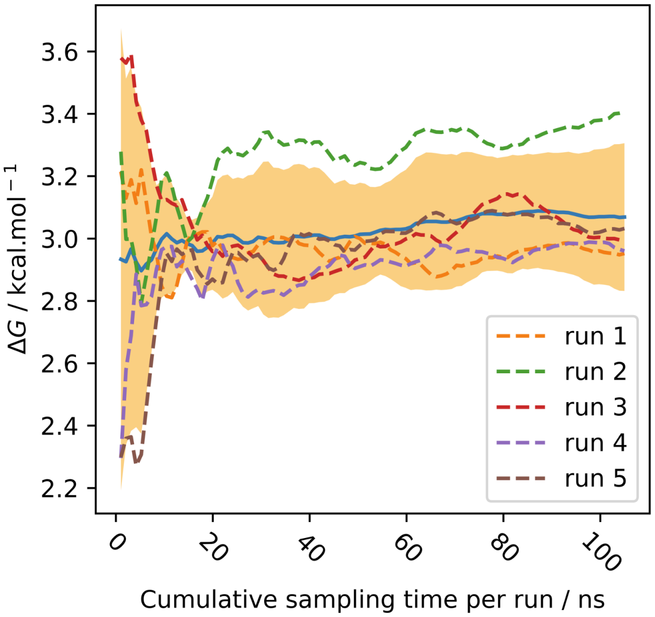

Figure S21: Convergence of the free energy of releasing all distance restraints other than the single strongest instance for M-Hand-R, with cumulative sampling time per window. Shaded area shows the 95% confidence interval and the solid blue line shows the mean.

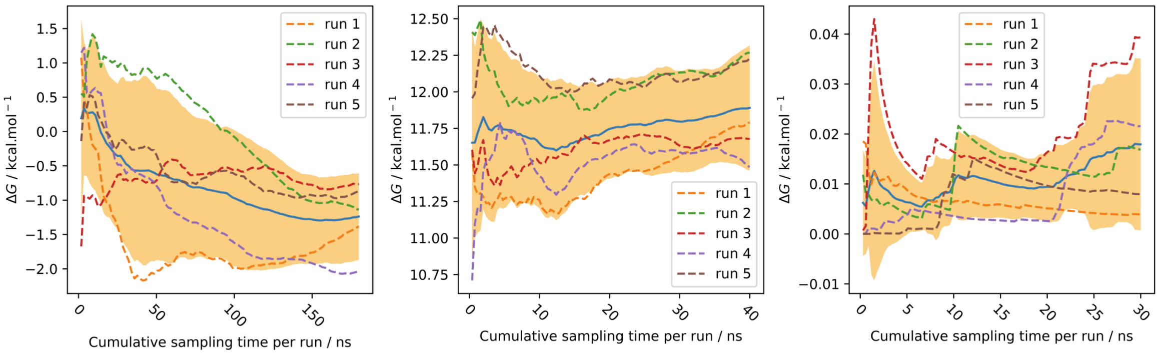

Figure S22: Convergence of the bound leg simulations for M-Hand with cumulative sampling time per window. From left to right: the vanish, discharge, and restrain stages. Shaded area shows the 95% confidence interval and the solid blue line shows the mean.

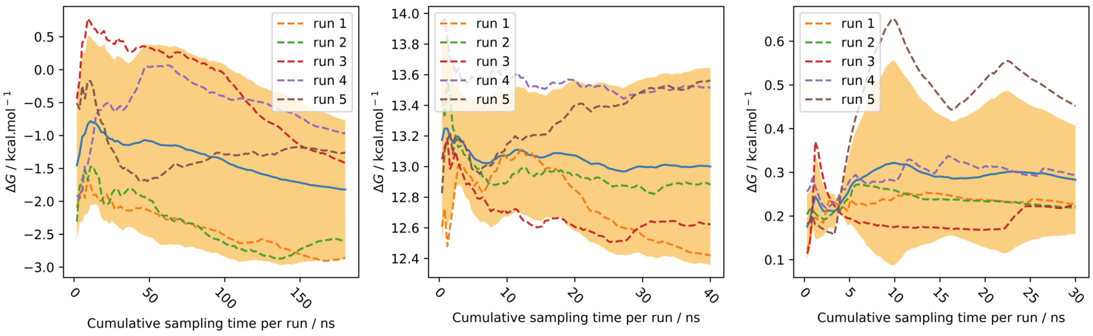

Figure S23: Convergence of the bound leg simulations for M-Hand-1 with cumulative sampling time per window. From left to right: the vanish, discharge, and restrain stages. Shaded area shows the 95% confidence interval and the solid blue line shows the mean.

S17 Overlap Matrices for Selected Multiple Distance Restraint Protocols

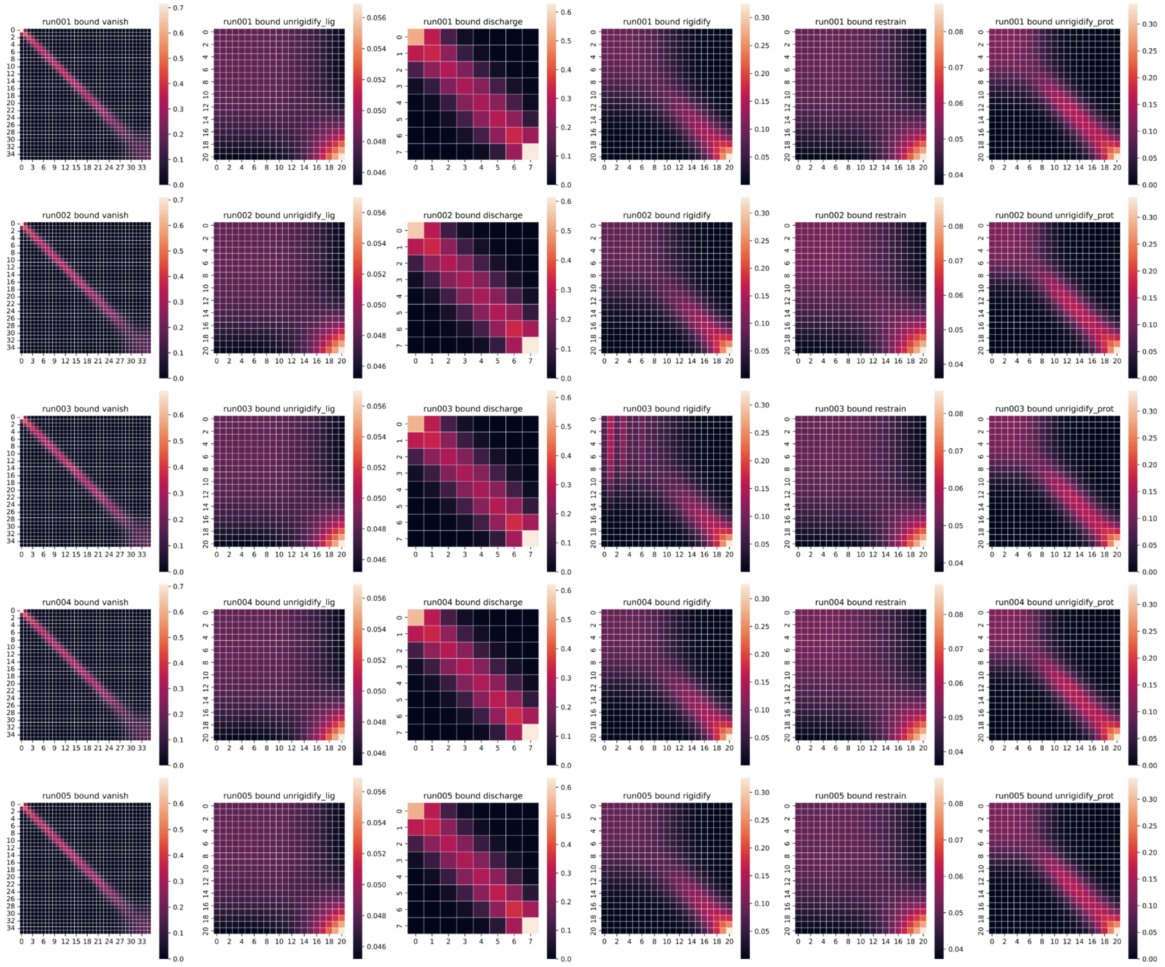

Figure S24: Overlap matricies for M-Rig

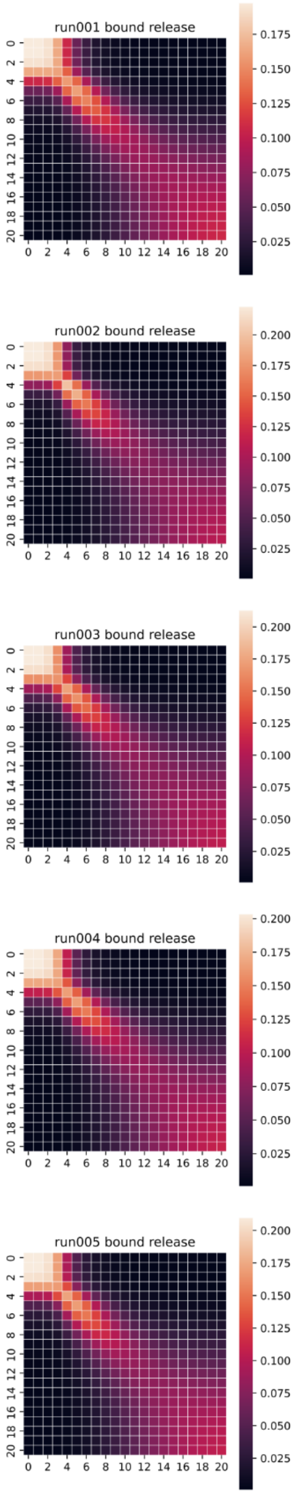

Figure S25: Overlap matricies for M-Hand-R

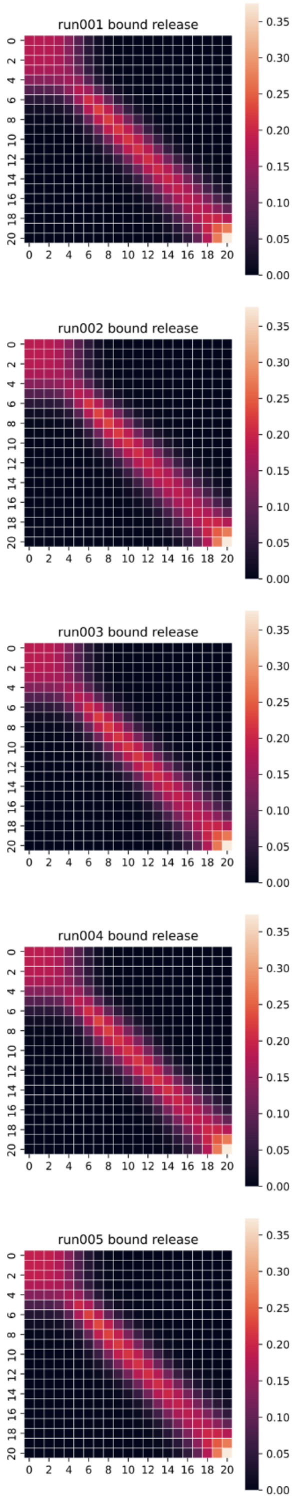

Figure S26: Overlap matricies for M-All-R

S18 Convergence of Free Energy of Preorganisation with Increasing Restraint Strength

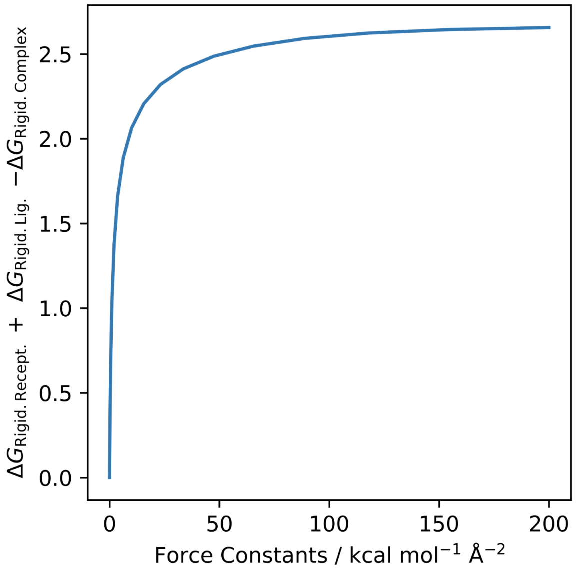

Figure S27: Convergence of $\Delta G_{\text{Rigid. Recept.}} + \Delta G_{\text{Rigid. Lig.}} - \Delta G_{\text{Rigid. Complex}} \approx \Delta G_{\text{Preorg.}}$ for M-Rig with respect to increasing strength of the intramolecular restraints, where the maximum strength of restraints was 200 kcal mol^{-1}. This is approximate because there are intermolecular interactions between the ligand and receptor when the restraints are introduced - this error could be eliminated by introduced the intramolecular restraints when the ligand is decoupled. A restraint strength of > 50 kcal mol^{-1} Å^{-2} would have been required to obtain result which was approximately converged with respect to increasing strength of the intramolecular restraints. For M-Rig, the intramolecular restraints of strength 75 kcal mol^{-1} produced $\Delta G_{\text{Rigid. Recept.}} + \Delta G_{\text{Rigid. Lig.}} - \Delta G_{\text{Rigid. Complex}} = 2.55$ kcal mol^{-1}, which was very similar to the result with 200 kcal mol^{-1} restraints (2.66 kcal mol^{-1}).

References

(1) Gilson, M.; Given, J.; Bush, B.; McCammon, J. The Statistical-Thermodynamic Basis for Computation of Binding Affinities: A Critical Review. *Biophys. J.* **1997**, *72*, 1047–1069.

(2) Boresch, S.; Tettinger, F.; Leitgeb, M.; Karplus, M. Absolute Binding Free Energies: A Quantitative Approach for Their Calculation. *J. Phys. Chem. B* **2003**, *107*, 9535–9551.

(3) Jorgensen, W. L.; Buckner, J. K.; Boudon, S.; Tirado-Rives, J. Efficient Computation of Absolute Free Energies of Binding by Computer Simulations. Application to the Methane Dimer in Water. *J. Chem. Phys.* **1988**, *89*, 3742–3746.

(4) Baumann, H. M.; Gapsys, V.; de Groot, B. L.; Mobley, D. L. Challenges Encountered Applying Equilibrium and Nonequilibrium Binding Free Energy Calculations. *J. Phys. Chem. B* **2021**, *125*, 4241–4261.

(5) Mobley, D. L.; Chodera, J. D.; Dill, K. A. On the Use of Orientational Restraints and Symmetry Corrections in Alchemical Free Energy Calculations. *J. Chem. Phys.* **2006**, *125*, 084902.

(6) Duboué-Dijon, E.; Hénin, J. Building Intuition for Binding Free Energy Calculations: Bound State Definition, Restraints, and Symmetry. *J. Chem. Phys.* **2021**, *154*, 204101.

(7) Shirts, M. R.; Chodera, J. D. Statistically Optimal Analysis of Samples from Multiple Equilibrium States. *J. Chem. Phys.* **2008**, *129*, 124105.

(8) Kirkwood, J. G. Statistical Mechanics of Fluid Mixtures. *J. Chem. Phys.* **1935**, *3*, 300–313.

(9) Wang, J.; Deng, Y.; Roux, B. Absolute Binding Free Energy Calculations Using Molecular Dynamics Simulations with Restraining Potentials. *Biophys. J.* **2006**, *91*, 2798–2814.

(10) Ebrahimi, M.; Hénin, J. Symmetry-Adapted Restraints for Binding Free Energy Calculations. *J. Chem. Theory Comput.* **2022**, *18*, 2494–2502.

(11) Alibay, I. IAlibay/MDRestraintsGenerator: MDRestraintsGenerator 0.1.0. Zenodo, 2021; `https://doi.org/10.5281/zenodo.4570556`.
